# Quantifying the relative contributions of bacterial and fungal communities to carcass decomposition using a quantitative microbiome profiling approach

**DOI:** 10.1038/s41522-025-00842-3

**Published:** 2025-11-17

**Authors:** Jun Zhang, Daijing Yu, Liuyaoxing Zhang, Tian Wang, Liwei Zhang, Jiangwei Yan

**Affiliations:** 1 School of Forensic Medicine, Shanxi Medical University, Jinzhong, Shanxi China; 2 Shanxi Key Laboratory of Forensic Medicine, Jinzhong, Shanxi China; 3 Shanxi Province Engineering Research Center of Forensic Identification, Jinzhong, Shanxi China

**Keywords:** Microbial communities, Environmental microbiology

## Abstract

Carcass microbial decomposition plays a vital role in global elemental cycling. However, bacterial and fungal absolute abundance dynamics, as well as their contributions to carcass decomposition, remain unclear. Here, the questions were investigated through quantitative microbiome profiling (QMP) and metabolomics. Within the first 14 days postmortem, microbial copies in grave soil and tissue increased by several orders of magnitude. Comparison of QMP with relative microbiome profiling (RMP) revealed strikingly different, even opposing successional trends for major phyla. Bacteria drove more metabolite variation than fungi in the decomposition. Co-occurrence networks revealed that key bacterial and fungal decomposers formed two distinct modules that were highly interconnected and significantly associated with carcass-derived metabolites, suggesting a synergistic relationship in the breakdown of organic matter. Notably, using QMP did not substantially enhance the accuracy of postmortem interval estimation. Collectively, our findings provide critical insights into microbial ecological dynamics during carcass decomposition.

## Introduction

Microbial decomposition of organic matter is a fundamental ecological process that underpins essential functions such as soil respiration and plant productivity ^1^. While the decomposition of plant litter, a major component of dead organic matter, has been extensively studied across diverse ecosystems^2–5^, the decomposition of animal remains represents an equally important yet comparatively underexplored process^6^. Unlike plant litter, which is primarily composed of cellulose, animal remains are rich in proteins and lipids^7^, leading to the generation of distinct metabolites, including polyamines, amines, benzene, and their derivatives^8^. Consequently, the microbial decomposer communities associated with animal remains differ markedly from those involved in plant litter degradation^9^. Previous studies have shown that ~20% of carbon and 2–3% of nitrogen from animal remains are released into the surrounding environment, thereby influencing soil fertility, ecosystem productivity, and associated biological communities^10,11^. Despite the ecological importance of animal decomposition, relatively few studies have addressed the microbial processes underlying this phenomenon.

Recent investigations have demonstrated that microbial succession during mammalian carcass decomposition exhibits certain patterns of consistency across host species and soil types^12,13^. Changes in the relative abundance of microbial taxa during this process have been extensively characterized, particularly in the context of postmortem interval (PMI) estimation, a topic of significant interest in forensic science. For instance, Metcalf et al. analyzed microbial communities in decomposing mouse carcasses and developed a predictive model for PMI, which was later extended to human cadavers^12,14^. Similarly, Liu et al. integrated postmortem microbial data with artificial neural networks (ANN), achieving PMI predictions with an accuracy of ~14.5 h over a 15-day period^15^.

These studies primarily focused on modeling the relationship between microbial succession and PMI, without examining the underlying metabolic activities of the microbial communities involved. As a result, they provide limited insight into the biogeochemical and nutrient cycling processes that accompany decomposition. In a more recent study, Burcham et al. monitored the microbiomes of 36 terrestrial human cadavers across three geographic locations and integrated these data with metabolomic profiles to explore links between microbial taxonomy and function^1^. Their findings advanced our understanding of cadaveric microbial ecology and underscored the importance of incorporating carbon and nutrient budgets into studies of carrion decomposition. Nevertheless, the specific metabolic functions of distinct microbial taxa during carcass decomposition remain poorly understood.

Mammalian carcasses provide nutrient-rich environments that support rapid microbial proliferation, leading to changes in both community composition and total microbial biomass^9^. However, most studies have relied on high-throughput sequencing to monitor shifts in community composition and relative abundance, offering limited information on absolute microbial abundance^14–16^.

Increasing evidence suggests that the quantification of total microbial biomass, or ‘absolute abundance’, provides a more accurate and comprehensive understanding of microbial dynamics than relative measures alone^17,18^. Quantitative microbiome profiling (QMP) has been proposed as a corrective approach to address biases inherent in relative microbiome profiling (RMP)^19,20^. For example, Tkacz et al. demonstrated that taxa appearing to decline in relative abundance may actually increase in absolute terms when total microbial load is considered^18^. Similarly, Nishijima et al. reported that microbial load is a key determinant of gut microbiome variation and is closely linked to host-associated factors, underscoring its importance in microbiome research^21^. The limitation of RMP approach may result in an incomplete comprehension of microbial succession patterns and obscure the ecological roles of specific taxa during decomposition.

To date, no study has comprehensively analyzed microbial succession based on absolute taxonomic abundance during carcass decomposition. This gap has hindered our understanding of the functional contributions of microbial taxa to decomposition and nutrient cycling. In this study, we investigated the succession of bacterial and fungal communities during the decomposition of Sprague-Dawley (SD) rat carcasses using QMP. By comparing QMP with RMP, we aim to provide a more accurate understanding of how the microbiology community truly changes over time during carcass decomposition. Key microbial decomposers were identified based on absolute abundance data. In parallel, we characterized metabolite dynamics using untargeted metabolomics and explored correlations between microbial succession and metabolic changes. Additionally, we evaluated and compared the predictive accuracy of PMI estimation models based on QMP, RMP, and metabolite data. Our findings aim to provide a more comprehensive understanding of the microbial metabolic processes and nutrient fluxes that govern carcass decomposition.

## Results

### Abundance and diversity of microbial communities during carcass decomposition

**Fig. 1:**
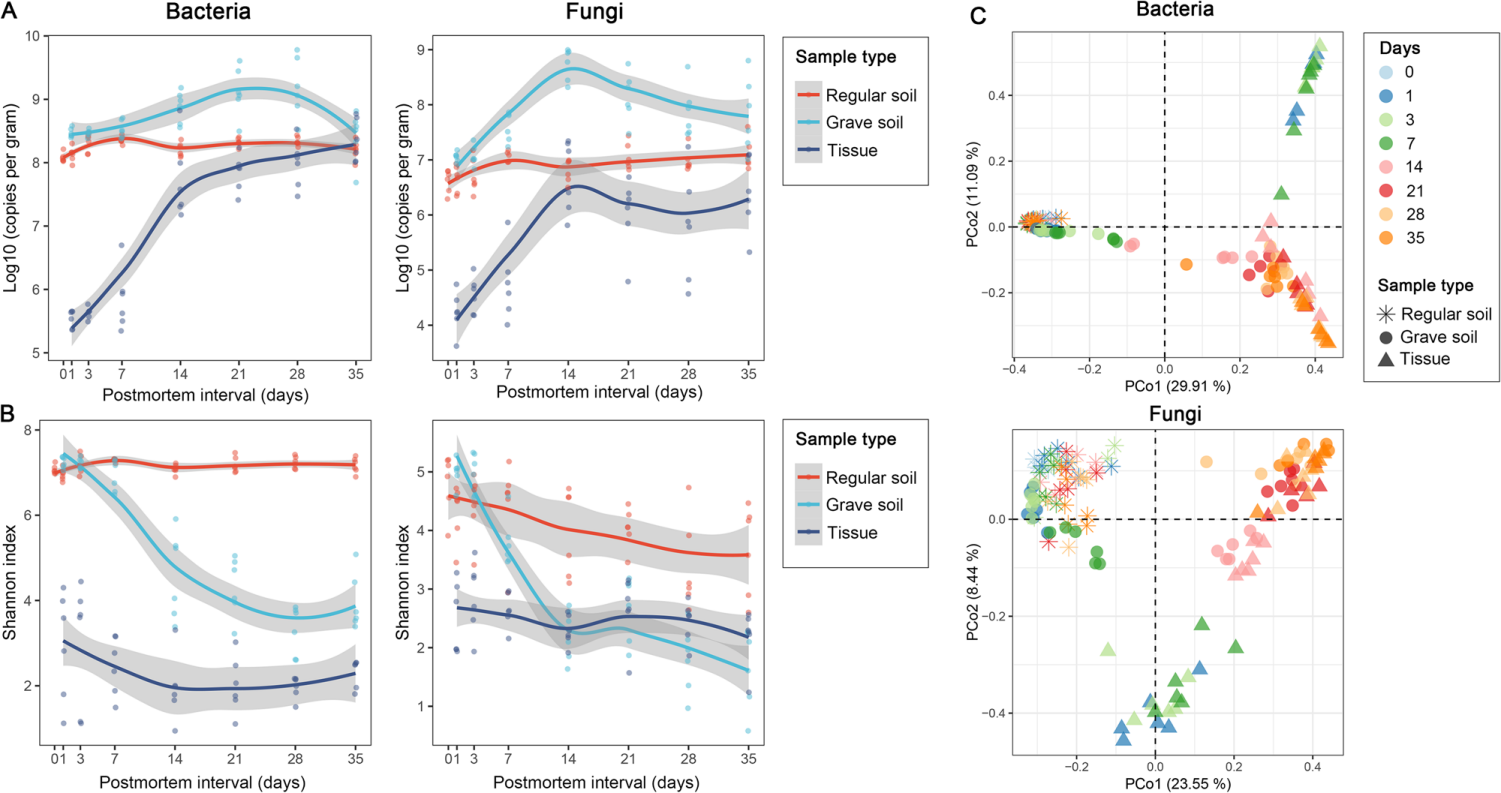
Changes in abundance and diversity of the quantitative microbiota during carcass decomposition. **A** Temporal dynamics of the absolute abundances of bacteria and fungi in soil, grave soil, and tissue samples. **B** Shannon diversity indices of microbial communities during decomposition. **C** Principal coordinates analysis (PCoA) based on Bray–Curtis dissimilarity illustrating compositional variation across sample types and postmortem intervals.

Overall, the copy number of bacterial 16S rRNA genes exceeded that of fungal ITS genes per gram of samples throughout the study period (Fig. 1A). In regular soil samples, the copies of 16S rRNA and ITS genes per gram remained relatively stable, whereas the most substantial fluctuations were observed in tissue samples. Rapid proliferation of both bacterial and fungal communities occurred during the first 14 days postmortem. Thereafter, bacterial growth slowed, while fungal populations declined over time in tissue. Initially, the copies of 16S rRNA and ITS genes per gram in grave soil were comparable to those in regular soil, but exhibited a transient increase followed by a subsequent decline (Fig. 1A).

Alpha diversity of regular soil bacterial communities remained relatively stable, whereas fungal alpha diversity gradually declined (Fig. 1B). In grave soil, a general downward trend in alpha diversity was observed for both bacterial and fungal communities. In tissue, alpha diversity for both domains declined within the first 14 days, followed by partial recovery thereafter (Fig. 1B).

Principal coordinates analysis (PCoA) revealed distinct successional patterns in microbial communities from both grave soil and tissue (Fig. 1C). Regular soil samples exhibited tight clustering, indicating relative temporal stability of the soil microbiome. Initially, microbial profiles of grave soil clustered closely with those of regular soil. By day 7, grave soil and tissue samples formed distinct clusters, but began to converge again by day 14, reflecting the progression of decomposition.

**Fig. 2:**
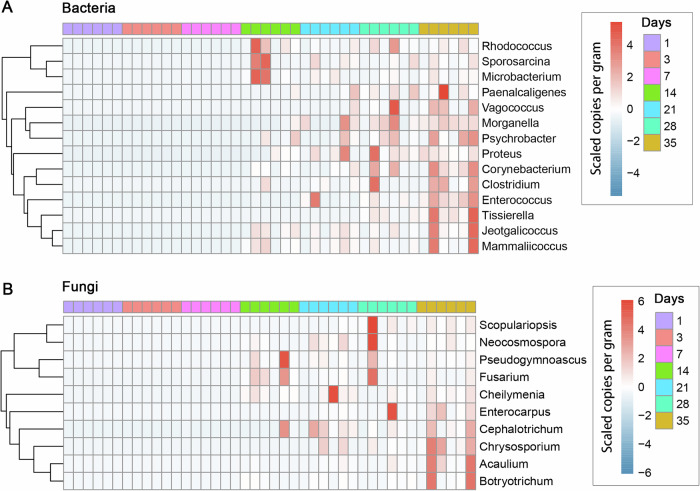
Abundance of identified key decomposers in tissue samples. Identification of key decomposers in tissue samples based on significant positive correlations (Spearman' *r* > 0.6, *P* < 0.001) between microbial abundance and postmortem interval. **A** Key bacterial decomposers in tissue samples. **B** Key fungal decomposers in tissue samples. The units represent scale-transformed gene copies per gram.

Fourteen bacterial and ten fungal genera were identified as key decomposers in carcass tissue (Fig. 2). These genera were significantly positively correlated with PMI, as evidenced by Spearman’s correlation coefficients > 0.6 (*P* < 0.001).

### A comparison of RMP and QMP methods in profiling microbial succession and interactions

**Fig. 3:**
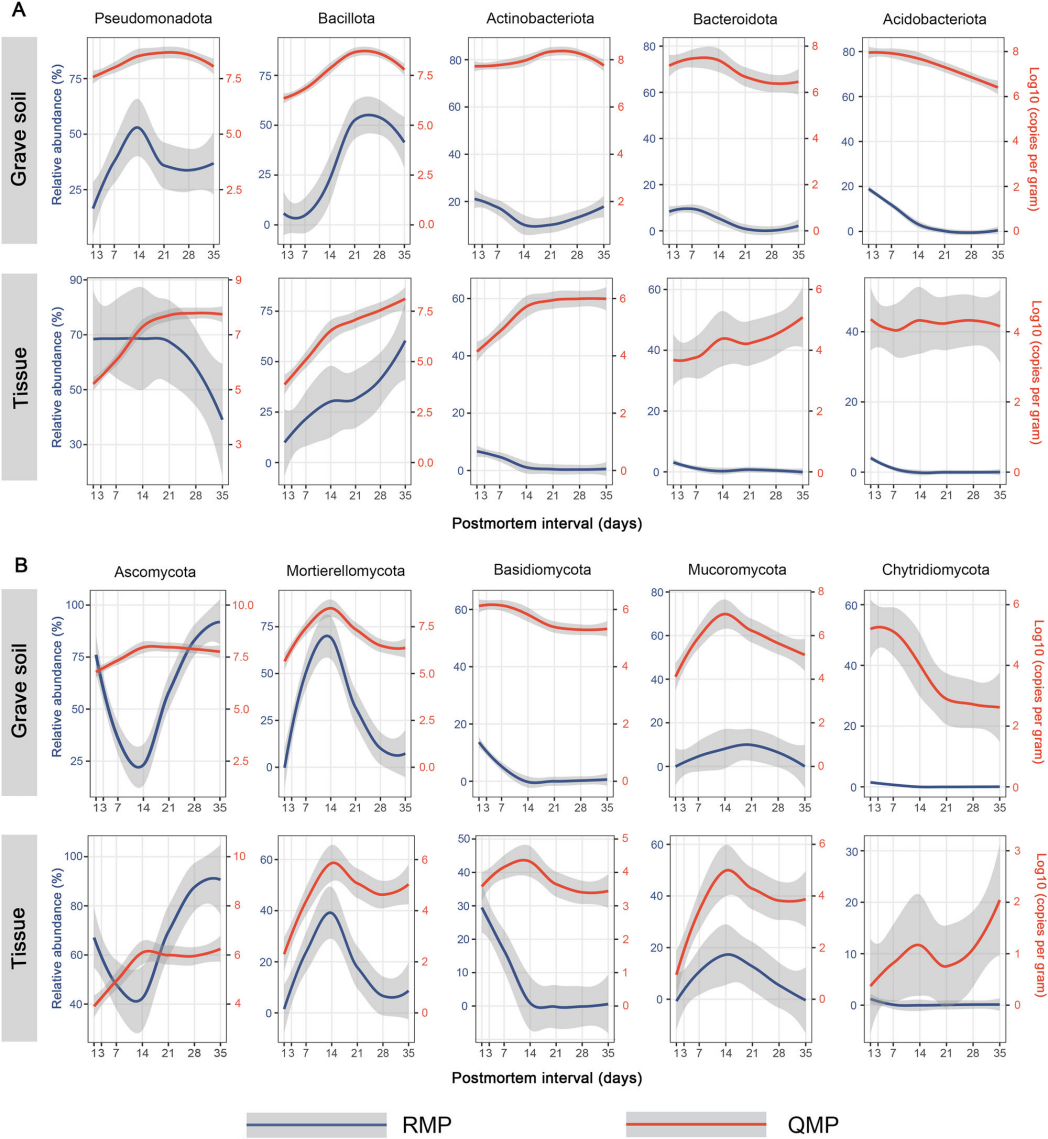
Comparisons of absolute and relative abundance trends for dominant phyla in grave soil and tissue samples. **A** The comparisons for dominant bacterial phyla. **B** The comparisons for dominant fungal phyla. Smoothed lines represent LOESS fits, with 95% confidence intervals indicated by the gray shaded area. The red line indicates absolute abundance, while the blue line denotes relative abundance.

Successional patterns of microbial communities derived from QMP and RMP data were compared at both phylum and genus levels. In regular soil samples, both absolute and relative abundances of bacterial dominant phyla remained relatively stable. While the abundances of the fungal dominant phyla varied in different time points, the change trends of both relative and absolute abundance remained largely the same (Supplementary Fig. S1). Therefore, the following analysis mainly focused on grave soil and tissue samples. In the two types of samples, several dominant phyla exhibited markedly different, and in some cases opposing, trends when profiled using QMP versus RMP (Fig. 3 and Supplementary Figs. S2–S5). For instance, *Pseudomonadota* displayed a decreasing trend in tissue samples based on RMP, whereas QMP revealed an increasing trend (Fig. 3A and Supplementary Fig. S3). Similarly, RMP indicated an initial decline followed by an increase in *Ascomycota*, whereas QMP revealed the opposite trend (Fig. 3B and Supplementary Figs. S4, S5). Contrasting patterns were also observed for *Actinobacteriota* in grave soil and tissue (Fig. 3A and Supplementary Figs. S2, S3), and *Basidiomycota* in tissue (Fig. 3B and Supplementary Fig. S5), when comparing RMP and QMP results.

The test assesses whether there is a statistically significant change in the abundance of the top 100 genera between samples collected before day 7 and those collected after day 14, using the Wilcoxon rank-sum test with Benjamini–Hochberg correction. Then, a comparison was conducted to determine the consistency of the results derived from QMP and RMP data (Supplementary Fig. S6). This analysis revealed 21 bacterial and 31 fungal genera in grave soil, and 69 bacterial and 15 fungal genera in tissue, that exhibited contrasting trends between RMP and QMP. These results underscore substantial differences in microbial succession profiling as characterized by the RMP approach in comparison to the QMP approach.

To quantify microbial succession rates, we employed time-decay models using both QMP and RMP data (Supplementary Fig. S7). Both approaches revealed significant (*P* < 0.05) linear time-decay relationships with PMI. Succession rates, represented by the slope of the regression line, were greater in bacterial and fungal communities of tissue when using QMP data. In grave soil, fungal succession rates were similar between QMP and RMP, while RMP data yielded a higher succession rate for bacteria than QMP.

Genus-level co-occurrence networks were constructed to explore microbial interactions as inferred from QMP and RMP data (Supplementary Fig. S8). In general, grave soil exhibited more complex network structures than tissue. RMP networks appeared more complex than their QMP counterparts, except for the fungal networks in tissue (Supplementary Fig. S8A, B). The RMP approach overestimated numerous correlations: only 23 bacterial and 21 fungal correlations (accounting for 3.96% and 12.89%, respectively) identified by RMP in tissue overlapped with those detected using QMP (Supplementary Fig. S8C, D). While the overlapping proportions between QMP and RMP reached at 53.88% and 40.48% in grave soil bacterial and fungal networks, respectively.

### Changes in metabolite abundance during carcass decomposition

**Fig. 4:**
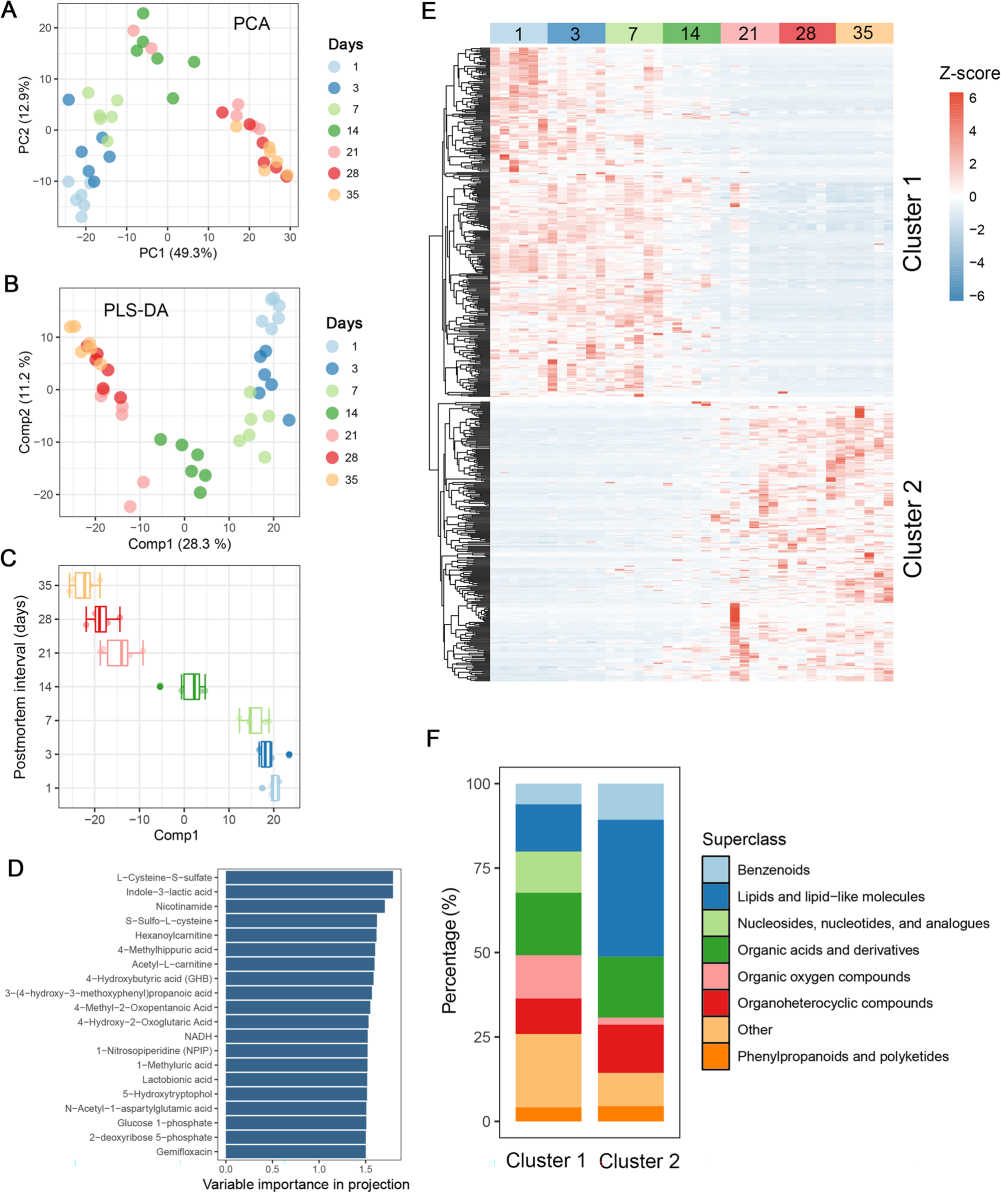
Changes in the metabolite profiles from tissue during decomposition of carcasses. **A** The principal component analysis (PCA) score plot of metabolites of carcass tissue across different postmortem intervals. **B** The partial least squares discriminant analysis (PLS-DA) score plot was constructed using metabolite profiles derived from carcass tissue, with the discriminatory variable defined by different postmortem intervals (day 1, day 3, day 7, day 14, day 21, day 28 and day 35). **C** Value of the first axis (comp1) generated from the PLS-DA at different postmortem intervals. Comp1 explained 28.3% of the variation among the seven groups. **D** Importance of metabolites ranked by variable importance in projection (VIP > 1.5 and *P* < 0.05). **E** Heatmap of metabolites significantly correlated to postmortem intervals are divided into two clusters (positive and negative relationships) based on hierarchical clustering. **F** Summary of pathway analysis at superclass level for each cluster.

A time-course untargeted metabolomic analysis was conducted to examine changes in tissue metabolite profiles during carcass decomposition (Fig. 4). Following data preprocessing, a total of 2614 metabolic features were identified (Supplementary Table S1). Of these, 576, 934, and 397 metabolites were annotated by the KEGG pathway, HMDB, and LIPID MAPS databases, respectively. Principal component analysis (PCA) and partial least squares discriminant analysis (PLS-DA) demonstrated that the samples grouped according to PMI. Specifically, the first principal component of the PCA accounted for 49.30% of the total variance (Fig. 4A), whereas the first component of the PLS-DA explained 28.30% of the variance (Fig. 4B). Samples collected before day 7 exhibited markedly different metabolic profiles compared to those collected after day 14 (Fig. 4A–C). Metabolites with a variable importance in projection (VIP) score greater than 1.5 were considered to be major contributors to group separation across time points. Twenty such metabolites were identified (Fig. 4D), with L-cysteine-S-sulfate, indole-3-lactic acid, and nicotinamide emerging as the primary discriminators.

Spearman’s correlation analysis identified 557 metabolites that were significantly correlated with PMI (*P* < 0.001, |r| > 0.6). Of these, 313 metabolites (Cluster 1) were negatively correlated with PMI, while 244 (Cluster 2) showed a positive correlation (Fig. 4E). Organic acids and their derivatives constituted 18.53% of metabolites in Cluster 1. In contrast, lipids and lipid-like molecules accounted for 40.57% of metabolites in Cluster 2 (Fig. 4F).

### Associations between microbiota and metabolites

**Fig. 5:**
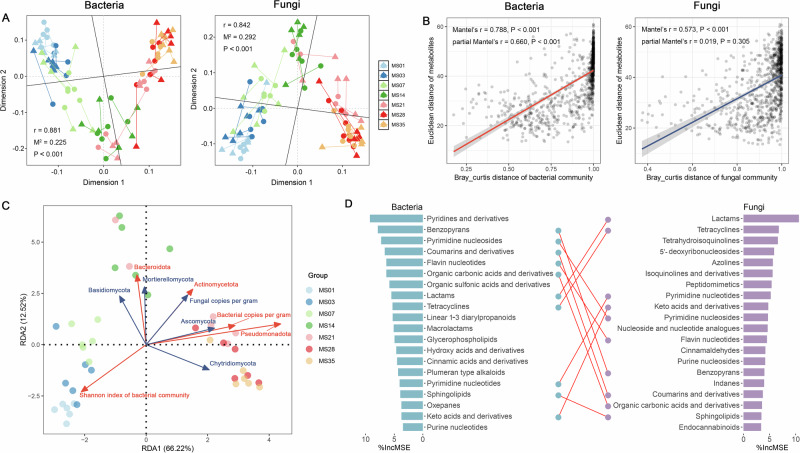
Correlations between microbiota and metabolites. **A** Procrustes analyses of the correlation between metabolites and absolute abundance for bacteria and fungi. **B** Mantel and partial mantel tests were utilized to explore the correlations between microbiota and metabolites. **C** Redundancy analysis (RDA) was employed to elucidate the contributions of the copies of 16S rRNA and ITS genes per gram, alpha diversity, and phyla abundance to the production of metabolites. **D** The intersection of metabolites most relevant (top 20) to absolute microbiota abundances of bacteria and fungi through random forest algorithm.

Procrustes analysis revealed a strong correlation between microbial communities and metabolites in decomposing tissue (bacteria: *r* = 0.881, M² = 0.225; fungi: *r* = 0.842, M² = 0.292; both *P* < 0.001), with low residuals (0.061 ± 0.041 for bacteria; 0.069 ± 0.047 for fungi) (Fig. 5A and Supplementary Fig. S9). These associations were further supported by Mantel and partial Mantel tests (Fig. 5B). The bacterial community showed a stronger independent correlation with metabolites (*r* = 0.660, *P* < 0.001) than the fungal community (*r* = 0.019, *P* = 0.305) when controlling for the influence of the other. Redundancy analysis (RDA) identified the copies of 16S rRNA and ITS genes per gram, along with the abundances of three bacterial and four fungal phyla, as significant contributors to metabolite variation (Fig. 5C). Among these, *Pseudomonadota* exerted the most prominent influence.

A random forest model was applied to identify metabolites most closely associated with microbial abundances. Pyridines and derivatives, benzopyrans, and pyrimidine nucleosides were predominantly associated with bacterial communities (Fig. 5D). In contrast, fungal communities were most strongly linked to lactams, tetracyclines, and tetrahydroisoquinolines. Notably, ten metabolite classes were shared among the top 20 most relevant to both bacterial and fungal communities, suggesting cooperative interactions in specific metabolic pathways during decomposition.

**Fig. 6:**
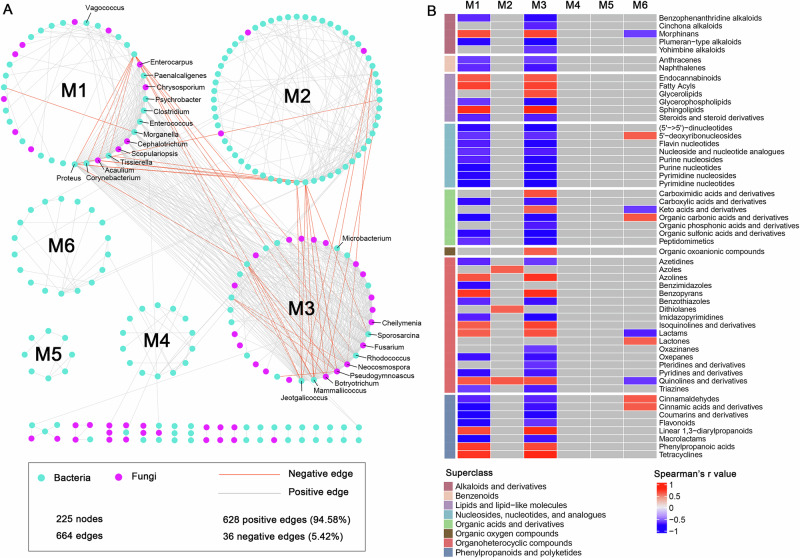
Microbial co-occurrence relationships and microbe-metabolite interactions in tissue of carcasses. **A** A co-occurrence network constructed with bacterial and fungal genera. Those nodes that were annotated is the key decomposers mentioned in the previous text. **B** Spearman’s correlations between modules from the co-occurrence network and metabolites. The red and blue grids indicate a significant (*P* < 0.05) positive and negative correlation, respectively. The gray grids indicate that the correlation is not significant.

To further explore microbial interactions, a co-occurrence network was constructed based on the absolute abundances of bacterial and fungal genera (Fig. 6). The network consisted of 225 nodes (170 bacterial and 55 fungal genera) and 664 edges (628 positive, 36 negative), with a modularity of 0.52, indicative of a modular structure (Supplementary Table S2). Six modules were identified, each comprising more than six nodes. Modules 4, 5, and 6 were composed exclusively of bacterial genera; Modules 1 and 2 were bacterial-dominated; and Module 3 was fungal-dominated. All key microbial decomposers (those nodes that were annotated with text) were distributed across Modules 1 and 3 (Fig. 6A). Spearman’s correlation analysis revealed significant associations between 54 metabolite classes and specific modules, with Module 1 and 3 showing the strongest correlations (Fig. 6B).

### PMI prediction using random forest models

**Fig. 7:**
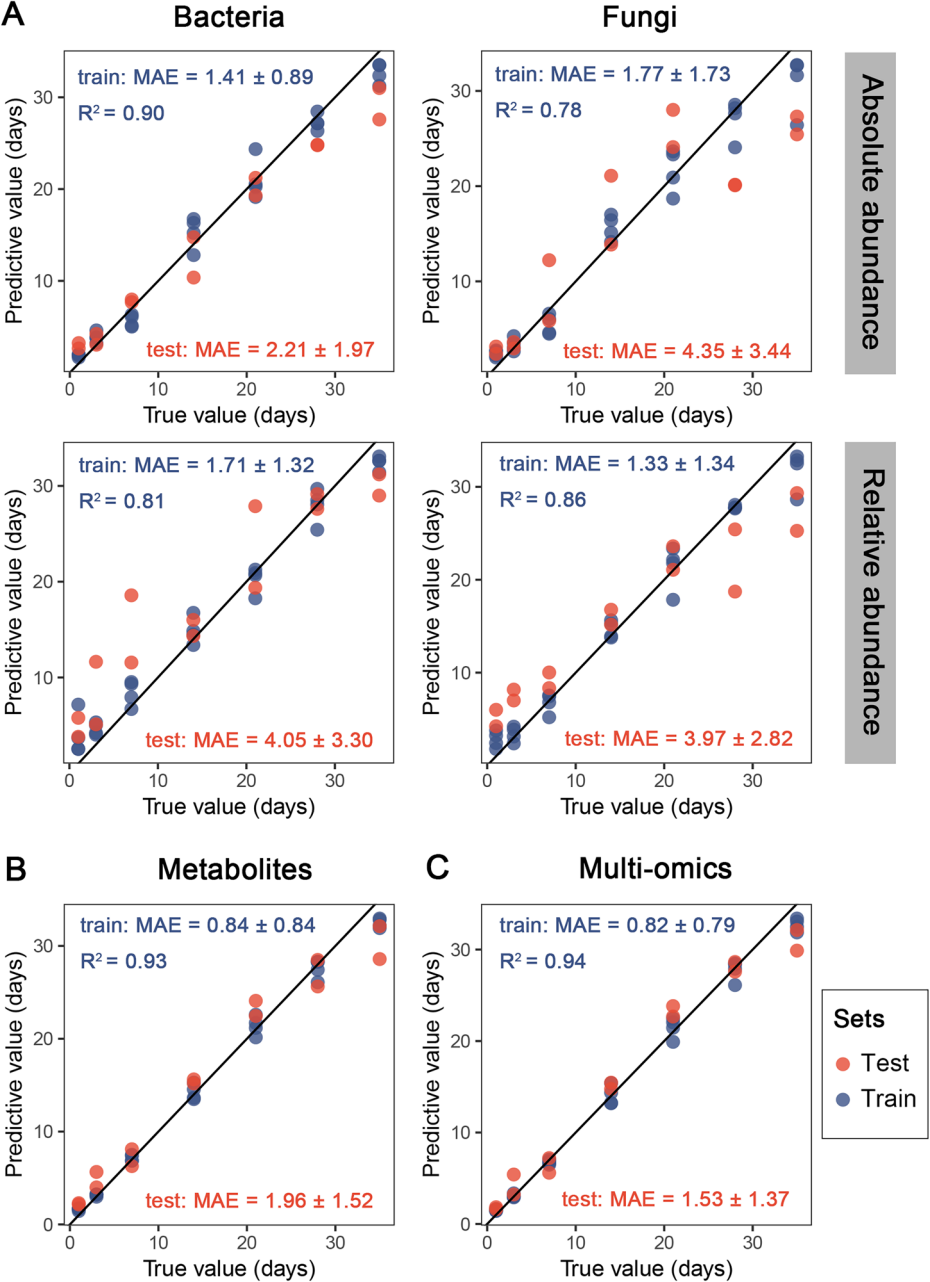
The predictive accuracies of PMI estimation performed with optimal biomarkers in tissue. **A** The prediction was conducted using absolute abundance and relative abundance of optimal biomarkers for bacteria and fungi, respectively. **B** The prediction was conducted using metabolic optimal biomarkers. **C** The prediction was conducted using multi-omics including metabolites and absolute abundances of bacterial and fungal communities.

Random forest algorithms were used to develop PMI prediction models based on absolute and relative microbial abundances at the amplicon sequence variant (ASV) level from grave soil and tissue. In addition, PMI prediction models were developed using metabolites and multi-omics data (including bacterial and fungal QMP data alongside metabolites) of training set. Biomarker sets were optimized via 10-fold cross-validation with five repetitions (Supplementary Figs. S10–S13), and the importance of each feature was assessed based on the increase in mean decrease accuracy (IncMSE) in the random forest model (Supplementary Table S3). The models were constructed using the optimal biomarkers, and their predictive performance was evaluated by inputting the training and testing sets into the models, respectively. The most models achieved lower mean absolute error (MAE) values and higher coefficient of determination (R²) values compared to those developed from the entire dataset (Supplementary Figs. S14, S15). Overall, MAEs derived from QMP and RMP data were comparable. The MAEs were similar when using bacterial and fungal data of grave soil samples (Supplementary Fig. S16), whereas in tissue, bacterial QMP data yielded the most accurate predictions for test sets (Fig. 7A). Metabolite-based PMI predictions using tissue-derived data achieved lower MAEs (training: 0.84 ± 0.84; testing: 1.96 ± 1.52) compared to microbiota-based models (Fig. 7B). Multi-omics data produced the most accurate PMI estimates (training: MAE = 0.82 ± 0.79; testing: MAE = 1.53 ± 1.37) (Fig. 7C).

## Discussion

This study characterized the succession of bacterial and fungal communities associated with decomposing carcasses using QMP approach and examined the correlations between microbial dynamics and metabolite changes during tissue decomposition. To our knowledge, this is one of the first studies to investigate microbial shifts in carcasses using a high-throughput quantitative methodology. The QMP approach offers a distinct advantage over RMP by mitigating compositionality effects, particularly relevant in processes marked by large fluctuations in microbial load, such as carcass decomposition^20^.

The regular soil, grave soil, and tissue samples analyzed here represent an effective gradient for assessing the differences between RMP and QMP. Among these, tissue samples exhibited the most pronounced variation in the copy numbers of 16S rRNA and ITS genes per gram expanding rapidly within 14 days postmortem. Several dominant phyla increased across multiple orders of magnitude, yet their successional patterns differed markedly when analyzed by RMP versus QMP. For instance, *Pseudomonadota*, *Actinobacteriota*, *Bacteroidota*, *Ascomycota*, and *Basidiomycota* displayed contrasting trends between the two approaches. Prior studies have reported a persistent decline in the relative abundance of *Actinobacteriota* in decomposing mouse organs^15^ and rat skin^16^. In contrast, our QMP-based analysis revealed these taxa as actively expanding during decomposition. Numerous studies have demonstrated that *Pseudomonadota* (syn. *Proteobacteria*) are associated with decomposition and increase in abundance in response to decomposing organic matter in initial stage^22–25^. However, studies on the decomposition of animal or human carcasses using the RMP method have shown that *Pseudomonadota* dominate the early decomposition, after which they are replaced by *Bacillota* (syn. *Firmicutes*)^15,16,26,27^. Our results based on the QMP method indicated that the population size of *Pseudomonadota* increased rapidly during the early stages of decomposition and remained relatively stable in the later stages. This suggests that *Pseudomonadota* may continue to play an important role even in the later stages of decomposition. We identified 14 bacterial and 10 fungal genera as key microbial decomposers significantly associated with PMI. Several of these taxa have been reported previously, including *Sporosarcina*, *Paenalcaligenes*, *Vagococcus*, *Morganella*, *Psychrobacter*, *Proteus*, *Tissierella*, *Enterococcus*, and *Clostridium*^1,14,28–30^, as well as fungal genera such as *Scopulariopsis*, *Pseudogymnoascus*, *Fusarium*, and *Chrysosporium*^31–33^. However, the abundance trajectories of many bacterial genera observed using QMP differed substantially from those inferred by RMP. For example, *Microbacterium* was previously reported to decrease in relative abundance during decomposition^34^, yet our data identified it as a key decomposer, highlighting how reliance on RMP may obscure the true ecological roles of certain taxa.

Our findings align with those of Feng et al., who reported that RMP approach was prone to overestimate or miss positive co-occurrence relationships found using RMP approach in chicken gut microbiota^35^. In our study, RMP approach substantially overestimated positive co-occurrence relationships found using the QMP approach among bacteria and underestimated those among fungi in tissue samples. These differences of positive relationships were less pronounced in grave soil, indicating that higher microbial load variation likely exacerbates differences between QMP and RMP. Similarly, Vandeputte et al. demonstrated that species interaction networks derived from co-occurrence data are vulnerable to compositionality effects arising from the use of relative abundance measurements^36^. The study revealed that the QMP network contained a far larger number of significantly co-varying genus pairs compared to the RMP network within the gut microbiota. These findings indicated that changes in absolute abundance are especially important for inferring and interpreting microbial co-occurrence relationships, and emphasized RMP’s limitations in accurately resolving microbial interactions. In addition, Vandeputte et al. noted that RMP can impede efforts to link microbiome features with quantitative traits, such as metabolite levels or physiological parameters, particularly under conditions of high microbial load variability^36^. In contrast, QMP reduces both false positives and false negatives in downstream microbiome analyses, thus improving the reliability of ecological and functional inferences^19,37^. These findings collectively underscore the necessity of adopting QMP over RMP for elucidating microbe–metabolite relationships during decomposition.

Distinct metabolite profiles were observed across different decomposition stages. Cluster 1 comprised metabolites that negatively correlated with PMI, whereas Cluster 2 included those that positively correlated with PMI. Nucleosides, nucleotides and analogues, and organic oxygen compounds were enriched in Cluster 1. These compounds are rapidly degraded during early decomposition^38^, likely due to oxygen depletion and cessation of aerobic respiration within tissues^39^. In contrast, Cluster 2 was dominated by lipids and lipid-like molecules. As decomposers preferentially metabolize labile, proteinaceous substrates, more recalcitrant lipid compounds accumulate over time^1^.

Bacterial and fungal heterotrophs are the principal microbial agents responsible for decomposition processes^40^. While the respective roles of bacterial and fungal communities in plant litter decomposition have been extensively studied, investigations that concurrently assess their contributions to mammalian carcass decomposition remain limited. In the present study, both the copy numbers of the 16S rRNA and ITS genes per gram, as well as alpha diversity were found to influence the metabolite composition of decomposing carcasses. Among the key decomposer phyla, *Pseudomonadota* and *Actinomycetota* (bacteria), along with *Ascomycota* and *Basidiomycota* (fungi), are known to play critical roles in organic matter degradation and are integral to carbon and nitrogen cycling^41,42^. Our findings confirmed that these phyla significantly affect the metabolite landscape during carcass decomposition.

Procrustes analysis and Mantel tests indicated that bacterial communities exhibited a stronger correlation with carcass-derived metabolites than fungal communities. Previous study has demonstrated that bacteria and fungi possess distinct substrate utilization profiles, with bacteria being efficient recyclers of nutrient-rich organic material and fungi specializing in the degradation of more recalcitrant, nutrient-poor substrates^40^. López-Mondéjar et al. further proposed that bacterial taxa with high nitrogen biomass preferentially decompose nitrogen-rich substrates, whereas fungi may be more efficient in utilizing taxa with lower nitrogen content^43^. Unlike plant litter, cadavers represent a highly concentrated nutrient source, characterized by a low carbon-to-nitrogen ratio and high water content^44^, which likely promotes rapid bacterial proliferation. The more efficient utilization of the abundant organic nutrients released from carcasses may explain the stronger association of bacterial communities with these metabolites.

We observed considerable overlap among the metabolites most strongly associated with bacterial and fungal communities. Co-occurrence network analyses revealed structured interactions within and between bacterial and fungal taxa, with species grouped within the same module hub showing close associations, indicative of potential cooperative interactions during decomposition^45^. Modules 1 and 3 contained both bacterial and fungal decomposers that were significantly correlated with various metabolites derived from carcasses. These results suggest that, despite functional partitioning between bacteria and fungi, a synergistic relationship underpins their contributions to decomposition. This is consistent with findings by Burcham et al., who reported that bacteria and fungi collaboratively degrade cadaveric lipids, proteins, and carbohydrates via resource partitioning and cross-feeding mechanisms^1^. Fungal exoenzymes, which facilitate the breakdown of recalcitrant substrates, may generate intermediates accessible to bacteria, supporting interdomain trophic interactions^46^. Such cooperative dynamics, previously reported in soil systems between *Ascomycota* and bacterial phyla such as *Actinobacteria* and *Pseudomonadota*^47^, were similarly observed during carcass decomposition in this study.

Although numerous studies have demonstrated the potential of microbial succession, combined with machine learning approaches, to estimate PMI with high accuracy^14–16^, interest in such studies appears to diminish when human cadaver data are not included. The objective of the current study was not to revisit this issue, but to evaluate whether QMP or multi-omics approaches could enhance predictive accuracy. The random forest algorithm was selected due to its demonstrated efficacy in mitigating overfitting by constructing individual trees^48,49^. In addition, optimal biomarkers were selected based on their feature importance in the random forest model. The models constructed with the optimal set achieved lower MAE values and higher R^2^ values compared to those derived from the entire dataset. This suggests that the optimization process enhanced the performance of the model. Our results indicate that replacing RMP with QMP does not consistently improve predictive performance. While the integration of multi-omics data yielded slightly higher accuracy compared to microbial or metabolite data alone, the improvement was modest. Given the increased cost associated with QMP and multi-omics techniques, we recommend the continued use of RMP for PMI prediction applications.

This study provides a comprehensive analysis of the dynamic shifts in microbial communities during carcass decomposition using a QMP approach. The results indicate that QMP and RMP exhibit substantial differences in their interpretation of microbial succession and the interactions involved in decomposition. When used together, the two distinct methods can provide a more comprehensive insight into the temporal dynamics of differential microbial abundance during the decomposition process. The study also underscores the distinct contributions of bacteria and fungi to metabolite production throughout the decomposition process. Several limitations should be acknowledged. Although the estimated copy number per gram can provide a partial indication of microbial load, it should not be directly interpreted as an exact quantification of microbial abundance due to variation in ribosomal operon copy numbers across different taxa. Accurate microbial load estimates require some type of physical assessment of microbial cells^36^. Environmental variables such as soil type and climatic conditions were not considered, potentially affecting the generalizability of the results. Moreover, the observed associations between microbial taxa and metabolites were solely derived from association analyses. Future studies integrating microbial and biogeochemical analyses, including stable isotope tracing, are necessary to validate these associations and elucidate causal relationships. Despite these limitations, the present study advances our understanding of microbial ecological processes during carcass decomposition and highlights the value of QMP in accurately capturing microbial dynamics.

## Methods

### Experimental design and sample collection

A total of 42 SD rats (half male and half female), reared in cohousing conditions from birth, were obtained from the Experimental Animal Centre of Shanxi Medical University. All rats were humanely euthanized using CO_2_ gas followed by cervical dislocation. Carcasses were then buried at a depth of ~20 cm in soil with uniform composition to minimize disturbance from insects and scavengers. The study adhered to the ARRIVE guidelines and was conducted in accordance with the U.K. Animals (Scientific Procedures) Act of 1986 and associated ethical regulations. The study was approved by the Institutional Animal Care and Use Committee of Shanxi Medical University, China (No. 2023-064).

Carcasses were exhumed on days 1, 3, 7, 14, 21, 28, and 35 post-burial. At each time point, six carcasses (half male and half female) were recovered via destructive sampling and transported under low-temperature conditions to the laboratory. Soil adhering to the ventral surface of each carcass was gently brushed into sterile centrifuge tubes and designated as grave soil. Regular soil samples were collected concurrently at a 20 cm depth adjacent to the graves, including on the day of burial. For tissue sampling, the abdominal cavity was dissected using sterile surgical scissors, and abdominal muscle tissue was excised and stored in sterile tubes as tissue samples. Totally, 132 samples (48 regular soil, 42 grave soil and 42 tissue samples) were collected. All samples were stored at −80 °C until further processing.

### DNA extraction, sequencing, and microbial data analysis

Total genomic DNA was extracted from ~0.5 g of each soil sample or 0.2 g of each tissue sample using the FastDNA® SPIN Kit for Soil (MP Biomedicals, Santa Ana, CA, USA). The sample was precisely weighted and documented prior to the extraction of DNA (Supplementary Table S4). DNA concentration and integrity were assessed using a Qubit 3.0 fluorometer (Thermo Fisher Scientific, Waltham, MA, USA). Synthetic internal standard sequences (spike-ins) with known gradient copy numbers were added to each DNA extract prior to amplification^20^. The V4–V5 hypervariable regions of the 16S rRNA gene were amplified using primers 515F (5’-GTGCCAGCMGCCGCGG-3’) and 907R (5’-CCGTCAATTCMTTTRAGTTT-3’) for bacterial community profiling. For fungal profiling, the ITS2 region was amplified using primers ITS3-F (5′-GCATCGATGAAGAACGCAGC-3′) and ITS4-R (5′-TCCTCCGCTTATTGATATGC-3′). Amplicons were sequenced on the Illumina NovaSeq 6000 platform.

Raw sequencing data were processed using QIIME2 (version 2022.8)^50^. Adapter and primer sequences were trimmed using the *cutadapt* plugin (version 3.4), and quality filtering and ASV identification were performed using the *DADA2* plugin (version 1.26.0)^51^ with the following parameters: --dissi 0.00; --p-max-ee 2; --p-trim-left-f 0; --p-trim-left-r 0; --p-trunc-len-f 220; --p-trunc-len-r 220; --p-min-fold-parent-over-abundance 1.0; --p-chimera-method consensus. Taxonomic classification of ASVs was conducted with confidence threshold 0.7 using a Naive Bayes classifier trained on the SILVA database (v138.2) for bacteria^52^ and the UNITE database (v9.0) for fungi^53^. A standard curve for each sample (Supplementary Table S5) was generated based on the spike-in read counts versus the input spike-in copy number using the formula: *log(R*_*s*_*)* = *a* *×* *log(C*_*s*_*)* + *b*, where *R*_*s*_ and *C*_*s*_ represent the spike-in sequencing read counts and the input spike-in copy number of the sample, respectively; *a* and *b* represent the slope and the intercept, respectively. The absolute copy number of each ASV in each sample was then calculated by substituting the read counts of the corresponding ASV into the equation derived from the standard curve. Spike-in sequences were excluded from downstream analyses. Absolute copy numbers of ASVs were utilized for subsequent analyses in the QMP method. In contrast, for the rarefaction-based RMP method, samples were standardized to a same number of reads prior to downstream analyses.

Alpha diversity was assessed using the Shannon index. Beta diversity was visualized via PCoA based on Bray–Curtis distances using the *vegan* package in R^54^. Statistical comparisons of alpha diversity and taxonomic composition were performed using the Kruskal–Wallis or Wilcoxon rank-sum test with Benjamini–Hochberg correction. A time–decay relationship was modeled through linear regression between community similarity and the temporal interval across all sample pairs. Co-occurrence networks were constructed based on significant Spearman’s correlations (|r|> 0.6, *P* < 0.001) among the 100 most abundant genera. Network visualization was performed using Gephi^55^ and the *igraph* package in R^56^.

### Metabolic profiling of tissue and data analysis

Tissue samples (100 mg) from individual carcasses were homogenized under liquid nitrogen and resuspended in pre-chilled 80% methanol with vigorous vortexing. Samples were incubated on ice for 5 min, followed by centrifugation at 15,000 rpm for 20 min at 4 °C. The resulting supernatants were collected for untargeted metabolomic analysis. Metabolites were profiled using ultra-high-performance liquid chromatography–tandem mass spectrometry (UHPLC-MS/MS) on a Vanquish UHPLC system coupled to an Orbitrap Q Exactive™ HF mass spectrometer (Thermo Fisher Scientific, Germany)^57^. Data were acquired in both positive- and negative-ion modes to maximize metabolite coverage. Raw data were processed using Compound Discoverer 3.3 (Thermo Fisher Scientific, Germany) for peak alignment, detection, and quantification. Metabolite annotation was performed using the KEGG (https://www.genome.jp/kegg/pathway.html), HMDB (https://hmdb.ca/metabolites), and LIPID MAPS (http://www.lipidmaps.org/) databases.

The resulting metabolite feature table was sum-normalized and pareto-scaled. PCA was performed using the prcomp function in R base stats package based on metabolite feature table. PLS-DA was applied using the *MetaboAnalystR* package in R^58^ to evaluate metabolite differences across PMIs. VIP scores were calculated to rank metabolite contributions to group separation, with VIP > 1.5 and *P* < 0.05 used as selection criteria for significant PMI-associated metabolites. Spearman’s correlation analysis was performed to identify metabolites significantly associated with PMI (*P* < 0.001, |*r*|> 0.6), and results were visualized using the *pheatmap* package in R^59^.

### Microbiota–metabolite correlation analysis

Procrustes analysis was conducted to assess the concordance between microbiota and metabolite profiles based on principal component analysis using the *vegan* package in R^54^. Mantel and partial Mantel tests were applied to evaluate pairwise correlations between microbial and metabolite distance matrices. RDA was performed to investigate the contributions of the copies of 16S rRNA gene and ITS gene per gram, alpha diversity, and phylum-level abundance to metabolite variation. Random forest analysis was used to identify key metabolites associated with bacterial and fungal communities, ranked by IncMSE^60^. A genus-level microbial co-occurrence network integrating bacterial and fungal taxa was constructed based on significant Spearman’s correlations (|*r*|> 0.6, *P* < 0.001). Network visualization was carried out in Cytoscape^61^. Module eigengene analysis, representing the first principal component of each module, was employed to assess correlations between major modules and metabolite profiles^62^.

### PMI prediction analysis

Feature tables derived from QMP, RMP, and metabolomic datasets were used to construct random forest regression models for PMI prediction. A random stratified sampling method was used to divide the dataset into training and testing subsets at a ratio of 2:1. Specifically, for each time point, four samples were assigned to the training set, whereas the remaining two samples were allocated to the testing set. The number of trees was set to 1000. The importance of each feature in the random forest model was evaluated using IncMSE. IncMSE was calculated by permuting the data for each feature randomly while keeping the other features unchanged. Subsequently, the features were ranked according to their IncMSE values and selected through ten-fold cross-validation as the optimal set to minimize prediction error. A multi-omics predictive model was also developed, integrating QMP-derived bacterial and fungal profiles with tissue metabolomic data. Model performance was evaluated based on MAE and R² ^16^. In order to maintain the robustness of the results, the model was run 100 times to calculate the mean values of MAE and R^2^.

## Supplementary information

Supplementary Information

Supplementary TableS1.

Supplementary TableS3.

Supplementary TableS3.

Supplementary TableS4.

## Data Availability

All sequencing files resulting from the Illumina platform are publicly available in the Sequence Read Archive (SRA) of the National Center for Biotechnology Information (NCBI) database (bacteria 16S rRNA gene: accession no. PRJNA1251147; fungal ITS: accession no. PRJNA1251276). Metabolomic data is available at BIG Submission (BIG SUB, bioproject PRJCA038875, OMIX ID: OMIX009865).

## Code availability

The R code used in this study is available on GitHub (https://github.com/Jun2537/decomposition-microbiome.git).

## References

[1] Burcham, Z. M. et al. A conserved interdomain microbial network underpins cadaver decomposition despite environmental variables. *Nat. Microbiol.* **9**, 595–613 (2024). 38347104 10.1038/s41564-023-01580-y PMC10914610

[2] Wang, W. et al. Effects of mixed-species litter on bacterial and fungal lignocellulose degradation functions during litter decomposition. *Soil Biol. Biochem.* **141**, 107690 (2020).

[3] Cao, T. et al. Enlarging interface reverses the dominance of fungi over bacteria in litter decomposition. *Soil Biol. Biochem.* **198**, 109543 (2024).

[4] Purahong, W. et al. Life in leaf litter: novel insights into community dynamics of bacteria and fungi during litter decomposition. *Mol. Ecol.* **25**, 4059–74 (2016). 27357176 10.1111/mec.13739

[5] Tláskal, V. et al. Complementary roles of wood-inhabiting fungi and bacteria facilitate deadwood decomposition. *Msystems* **6**, 10–1128 (2021). 10.1128/mSystems.01078-20 PMC7901482 33436515

[6] Finley, S. J., Pechal, J. L., Benbow, M. E., Robertson, B. K. & Javan, G. T. Microbial signatures of cadaver gravesoil during decomposition. *Micro Ecol.* **71**, 524–529 (2016). 10.1007/s00248-015-0725-1 26748499

[7] Han, Q. et al. Temporal dynamics of the diazotrophic community during corpse decomposition. *Appl. Microbiol. Biotechnol.* **108**, 1–15 (2024). 39520567 10.1007/s00253-024-13329-6 PMC11550258

[8] Zhou, R. et al. Carcass decomposition influences the metabolic profiles and enriches noxious metabolites in different water types by widely targeted metabolomics. *Chemosphere* **269**, 129400 (2021). 33383254 10.1016/j.chemosphere.2020.129400

[9] DeBruyn, J. M., Keenan, S. W. & Taylor, L. S. From carrion to soil: microbial recycling of animal carcasses. *Trends Microbiol.* **33**, 194–207 (2025). 39358066 10.1016/j.tim.2024.09.003

[10] Carter, D. O., Yellowlees, D. & Tibbett, M. Cadaver decomposition in terrestrial ecosystems. *Naturwissenschaften* **94**, 12–24 (2007). 17091303 10.1007/s00114-006-0159-1

[11] Chowdhury, S., Kim, G.-H., Ok, Y. S. & Bolan, N. Effect of carbon and nitrogen mobilization from livestock mortalities on nitrogen dynamics in soil. *Process Saf. Environ.* **122**, 153–160 (2019).

[12] Metcalf, J. L. et al. Microbial community assembly and metabolic function during mammalian corpse decomposition. *Science* **351**, 158–62 (2016). 26657285 10.1126/science.aad2646

[13] Metcalf, J. L. Estimating the postmortem interval using microbes: Knowledge gaps and a path to technology adoption. *Forensic Sci. Int. Genet.* **38**, 211–218 (2019). 30448529 10.1016/j.fsigen.2018.11.004

[14] Metcalf, J. L. et al. A microbial clock provides an accurate estimate of the postmortem interval in a mouse model system. *Elife* **15**, e01104 (2013). 10.7554/eLife.01104 PMC3796315 24137541

[15] Liu, R. et al. Predicting postmortem interval based on microbial community sequences and machine learning algorithms. *Environ. Microbiol.* **22**, 2273–2291 (2020). 32227435 10.1111/1462-2920.15000

[16] Zhang, J. et al. Predicting the postmortem interval of burial cadavers based on microbial community succession. *Forensic Sci. Int. Genet.* **52**, 102488 (2021). 33667880 10.1016/j.fsigen.2021.102488

[17] Ji, B. W. et al. Quantifying spatiotemporal variability and noise in absolute microbiota abundances using replicate sampling. *Nat. Methods* **16**, 731–736 (2019). 31308552 10.1038/s41592-019-0467-y PMC7219825

[18] Tkacz, A., Hortala, M. & Poole, P. S. Absolute quantitation of microbiota abundance in environmental samples. *Microbiome* **6**, 110 (2018). 29921326 10.1186/s40168-018-0491-7 PMC6009823

[19] Tito, R. Y. et al. Microbiome confounders and quantitative profiling challenge predicted microbial targets in colorectal cancer development. *Nat. Med.* **30**, 1339–1348 (2024). 38689063 10.1038/s41591-024-02963-2 PMC11108775

[20] Maghini, D. G. et al. Quantifying bias introduced by sample collection in relative and absolute microbiome measurements. *Nat. Biotechnol.* **42**, 328–338 (2024). 37106038 10.1038/s41587-023-01754-3

[21] Nishijima, S. et al. Fecal microbial load is a major determinant of gut microbiome variation and a confounder for disease associations. *Cell* **188**, 222–236 (2025). 39541968 10.1016/j.cell.2024.10.022

[22] Yu, L. et al. An optimal combined slow-release nitrogen fertilizer and urea can enhance the decomposition rate of straw and the yield of maize by improving soil bacterial community and structure under full straw returning system. *Front. Microbiol.* **15**, 1358582 (2024). 38962118 10.3389/fmicb.2024.1358582 PMC11219627

[23] Zhou, Z., Wang, C. & Luo, Y. Effects of forest degradation on microbial communities and soil carbon cycling: a global meta-analysis. *Glob. Ecol. Biogeogr.* **27**, 110–124 (2018).

[24] Schneider, T. et al. Who is who in litter decomposition? Metaproteomics reveals major microbial players and their biogeochemical functions. *ISME J.* **6**, 1749–1762 (2012). 22402400 10.1038/ismej.2012.11 PMC3498922

[25] Bao, Y. et al. Important ecophysiological roles of non-dominant Actinobacteria in plant residue decomposition, especially in less fertile soils. *Microbiome* **9**, 84 (2021). 33827695 10.1186/s40168-021-01032-x PMC8028251

[26] Hyde, E. R., Haarmann, D. P., Lynne, A. M., Bucheli, S. R. & Petrosino, J. F. The living dead: bacterial community structure of a cadaver at the onset and end of the bloat stage of decomposition. *PloS One* **8**, e77733 (2013). 24204941 10.1371/journal.pone.0077733 PMC3813760

[27] Lauber, C. L. et al. Vertebrate decomposition is accelerated by soil microbes. *Appl. Environ. Micro* **80**, 4920–4929 (2014). 10.1128/AEM.00957-14 PMC4135757 24907317

[28] Cobaugh, K. L., Schaeffer, S. M. & DeBruyn, J. M. Functional and structural succession of soil microbial communities below decomposing human cadavers. *PloS One* **10**, e0130201 (2015). 26067226 10.1371/journal.pone.0130201 PMC4466320

[29] Johnson, A. P., Mikac, K. M. & Wallman, J. F. Thermogenesis in decomposing carcasses. *Forensic Sci. Int.* **231**, 271–277 (2013). 23890649 10.1016/j.forsciint.2013.05.031

[30] Zedi, S., Khandeparkar, R., Render, R. & Gracias, M. Bacterial succession during terrestrial and marine decomposition in fish and pig models to estimate post-mortem interval: death and decomposition. *Res. Sq.*10.21203/rs.3.rs-3604092/v1 (2023).

[31] Piepenbring, M. et al. Exploring the diversity of culturable fungi on corpses for forensic applications. *Mycol. Prog.* **24**, 1–15 (2025).

[32] Leite-Jr, D. et al. Action of fauna and flora on the cadaveric phenomena observed in the carcass of Sus scrofa (Linnaeus-Suidae) in the wild area Brazilian savannah of the central region-Brazil. *Forensic Res. Criminol. Int. J.* **7**, 185–199 (2019).

[33] Procopio, N. et al. Soil fungal communities investigated by metabarcoding within simulated forensic burial contexts. *Front. Microbiol.* **11**, 1686 (2020). 32793158 10.3389/fmicb.2020.01686 PMC7393272

[34] Liu, R. et al. Dissecting the microbial community structure of internal organs during the early postmortem period in a murine corpse model. *BMC Microbiol.* **23**, 38 (2023). 36765295 10.1186/s12866-023-02786-0 PMC9912631

[35] Feng, Y. et al. Quantitative microbiome profiling reveals the developmental trajectory of the chicken gut microbiota and its connection to host metabolism. *Imeta* **2**, e105 (2023). 38868437 10.1002/imt2.105 PMC10989779

[36] Vandeputte, D. et al. Quantitative microbiome profiling links gut community variation to microbial load. *Nature* **551**, 507–511 (2017). 29143816 10.1038/nature24460

[37] Vieira-Silva, S. et al. Statin therapy is associated with lower prevalence of gut microbiota dysbiosis. *Nature* **581**, 310–315 (2020). 32433607 10.1038/s41586-020-2269-x

[38] Kovács, Z. et al. Postmortem degradation of nucleosides in the brain: Comparison of human and rat brains for estimation of in vivo concentration of nucleosides. *J. Neurosci. Methods* **148**, 88–93 (2005). 16054224 10.1016/j.jneumeth.2005.04.012

[39] Costa, I. et al. Promising blood-derived biomarkers for estimation of the postmortem interval. *Toxicol. Res.* **4**, 1443–1452 (2015). 10.1039/c5tx00397k PMC6062304 30102300

[40] Fabian, J., Zlatanovic, S., Mutz, M. & Premke, K. Fungal–bacterial dynamics and their contribution to terrigenous carbon turnover in relation to organic matter quality. *ISME J.* **11**, 415–425 (2017). 27983721 10.1038/ismej.2016.131 PMC5270572

[41] Bastian, F., Bouziri, L., Nicolardot, B. & Ranjard, L. Impact of wheat straw decomposition on successional patterns of soil microbial community structure. *Soil Biol. Biochem.* **41**, 262–275 (2009).

[42] Wei, D., Wei, S., Peng, A., Yang, C. & Chen, C. Responses of bacterial communities in soils under winter wheat to nightly warming and nitrogen addition. *Agronomy* **12**, 1616 (2022).

[43] López-Mondéjar, R. et al. Decomposer food web in a deciduous forest shows high share of generalist microorganisms and importance of microbial biomass recycling. *ISME J.* **12**, 1768–1778 (2018). 29491492 10.1038/s41396-018-0084-2 PMC6018761

[44] Fu, X. et al. Fungal succession during mammalian cadaver decomposition and potential forensic implications. *Sci. Rep.* **9**, 12907 (2019). 31501472 10.1038/s41598-019-49361-0 PMC6733900

[45] Zhao, B., Xing, P. & Wu, Q. L. Interactions between bacteria and fungi in macrophyte leaf litter decomposition. *Environ. microbiol* **23**, 1130–1144 (2020). 33015932 10.1111/1462-2920.15261

[46] Wang, C. & Kuzyakov, Y. Mechanisms and implications of bacterial–fungal competition for soil resources. *ISME J.* **18**, wrae073 (2024). 38691428 10.1093/ismejo/wrae073 PMC11104273

[47] Zheng, W., Zhao, Z., Gong, Q., Zhai, B. & Li, Z. Responses of fungal–bacterial community and network to organic inputs vary among different spatial habitats in soil. *Soil Biol. Biochem.* **125**, 54–63 (2018).

[48] Halabaku, E. & Bytyçi, E. Overfitting in machine learning: a comparative analysis of decision trees and random forests. Intell Autom Soft Co. 39. 10.32604/iasc.2024.059429 (2024).

[49] Barreñada, L., Dhiman, P., Timmerman, D., Boulesteix, A. L. & Van Calster, B. Understanding overfitting in random forest for probability estimation: a visualization and simulation study. *Diagn. Progn. Res.* **8**, 14 (2024). 39334348 10.1186/s41512-024-00177-1 PMC11437774

[50] Bolyen, E. et al. Reproducible, interactive, scalable and extensible microbiome data science using QIIME 2. *Nat. Biotechnol.* **37**, 852–857 (2019). 31341288 10.1038/s41587-019-0209-9 PMC7015180

[51] Callahan, B. J. et al. DADA2: High-resolution sample inference from Illumina amplicon data. *Nat. Methods* **13**, 581–583 (2016). 27214047 10.1038/nmeth.3869 PMC4927377

[52] Quast, C. et al. The SILVA ribosomal RNA gene database project: improved data processing and web-based tools. *Nucleic Acids Res.* **41**, D590–D596 (2013). 23193283 10.1093/nar/gks1219 PMC3531112

[53] Abarenkov, K. et al. The UNITE database for molecular identification and taxonomic communication of fungi and other eukaryotes: sequences, taxa and classifications reconsidered. *Nucleic Acids Res.* **52**, D791–D797 (2024). 37953409 10.1093/nar/gkad1039 PMC10767974

[54] Oksanen, J. et al. The vegan package. *Community Ecol. Package* **10**, 631–637 (2007).

[55] Bastian, M., Heymann, S. & Jacomy, M. Gephi: an open source software for exploring and manipulating networks. In *Proceedings of the International AAAI Conference on Web and Social Media*. 361–362 (AAAI, 2009).

[56] Csardi, M. G. Package ‘igraph’. Last accessed 3 (9), 2013 (2013).

[57] Want, E. J. et al. Global metabolic profiling of animal and human tissues via UPLC-MS. *Nat. Protoc.* **8**, 17–32 (2013). 23222455 10.1038/nprot.2012.135

[58] Chong, J. & Xia, J. MetaboAnalystR: an R package for flexible and reproducible analysis of metabolomics data. *Bioinformatics* **34**, 4313–4314 (2018). 29955821 10.1093/bioinformatics/bty528 PMC6289126

[59] Kolde, R. & Kolde, M. R. Package ‘pheatmap’. R. package 1, 790 (2015).

[60] Jiao, S., Chen, W. & Wei, G. Core microbiota drive functional stability of soil microbiome in reforestation ecosystems. *Glob. Chang Biol.* **28**, 1038–1047 (2022). 34862696 10.1111/gcb.16024

[61] Cline, M. S. et al. Integration of biological networks and gene expression data using Cytoscape. *Nat. Protoc.* **2**, 2366–2382 (2007). 17947979 10.1038/nprot.2007.324 PMC3685583

[62] Zhang, B., Zhang, J., Liu, Y., Shi, P. & Wei, G. Co-occurrence patterns of soybean rhizosphere microbiome at a continental scale. *Soil Biol. Biochem.* **118**, 178–186 (2018).

