# Supplementary material for: Quantifying the relative contributions of bacterial and fungal communities to carcass decomposition using a quantitative microbiome profiling approach
Source: NPJ Biofilms Microbiomes. 2025 Nov 17;11:210. doi: 10.1038/s41522-025-00842-3 (PMC12624039; doi:10.1038/s41522-025-00842-3)
Supplement: Supplementary file 1 — Supplementary Information [file 41522_2025_842_MOESM1_ESM.pdf]

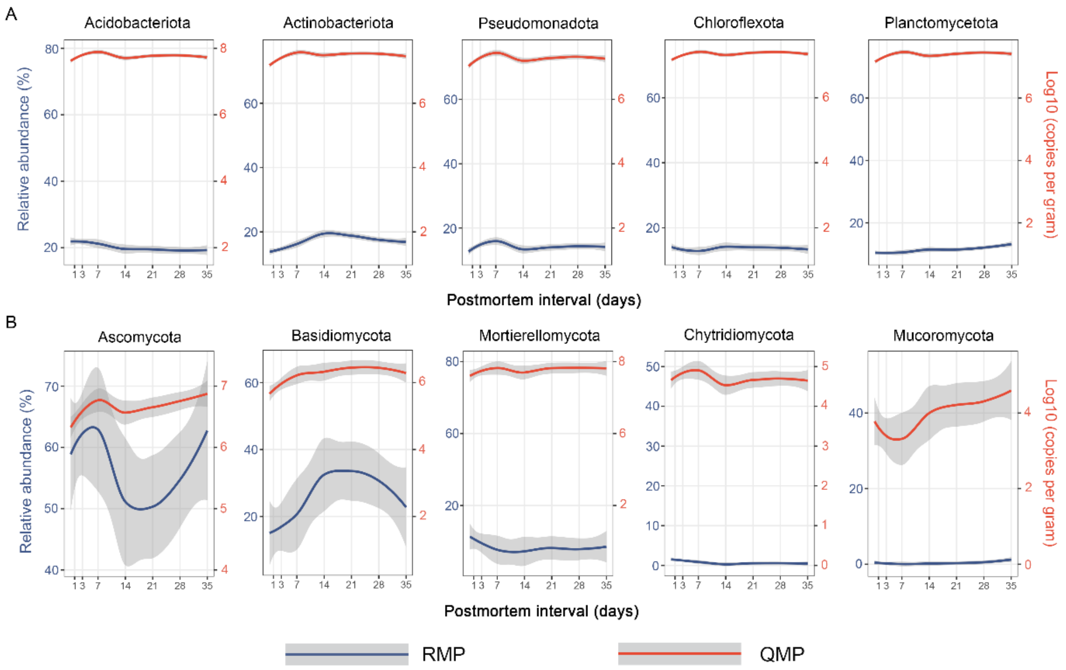

Fig. S1 Comparisons between absolute and relative abundance trends for dominant bacterial (A) and fungal (B) phyla in regular soil samples. Smoothed lines represent LOESS fits, with 95% confidence intervals indicated by the gray shaded area. The red line indicates absolute abundance, while the blue line denotes relative abundance.

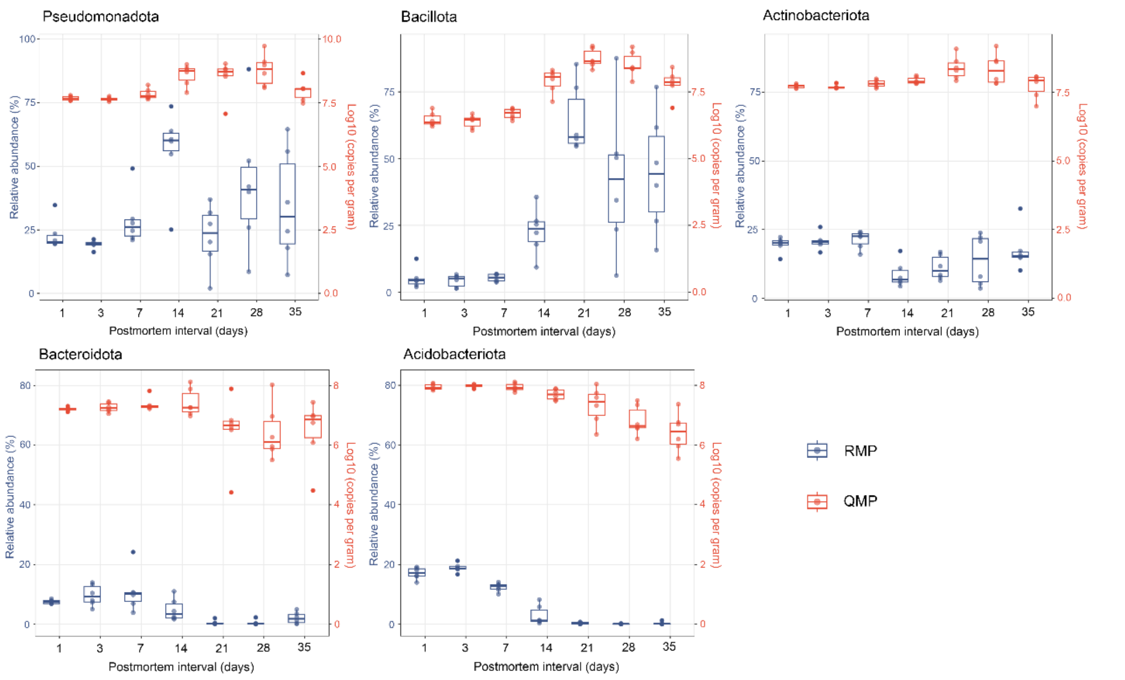

Fig. S2 A boxplot illustrates the comparisons of absolute and relative abundance trends of dominant bacterial phyla in grave soil samples. The red indicates absolute abundance, while the blue denotes relative abundance.

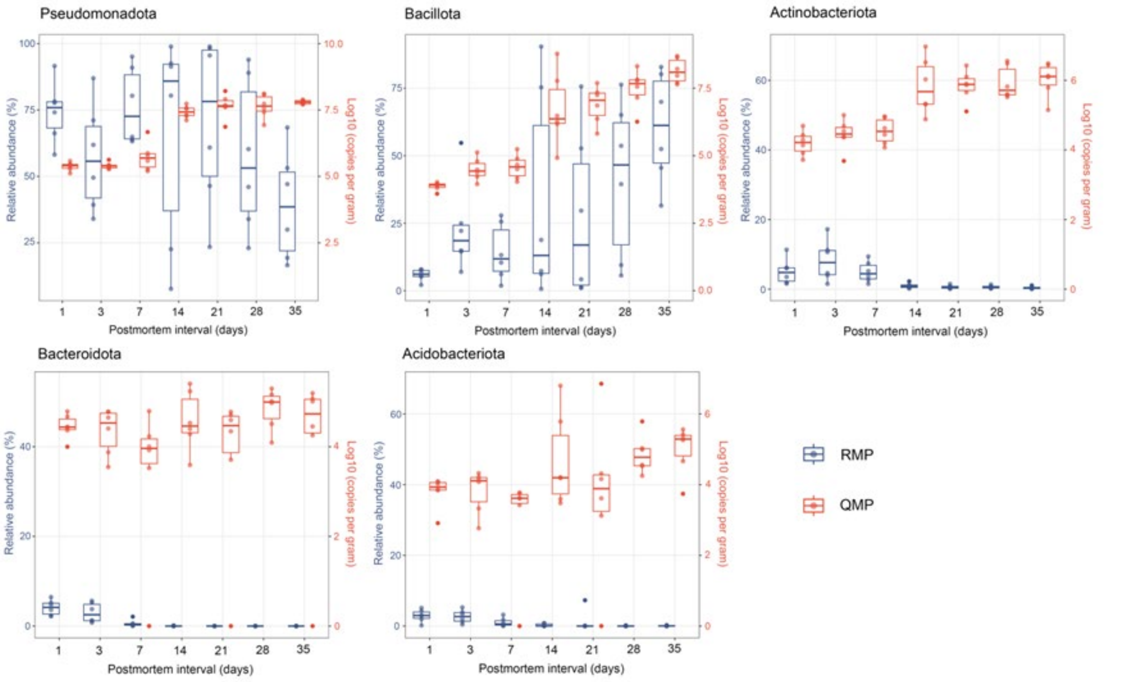

Fig. S3 A boxplot illustrates the comparisons of absolute and relative abundance trends of dominant bacterial phyla in tissue samples. The red indicates absolute abundance, while the blue denotes relative abundance.

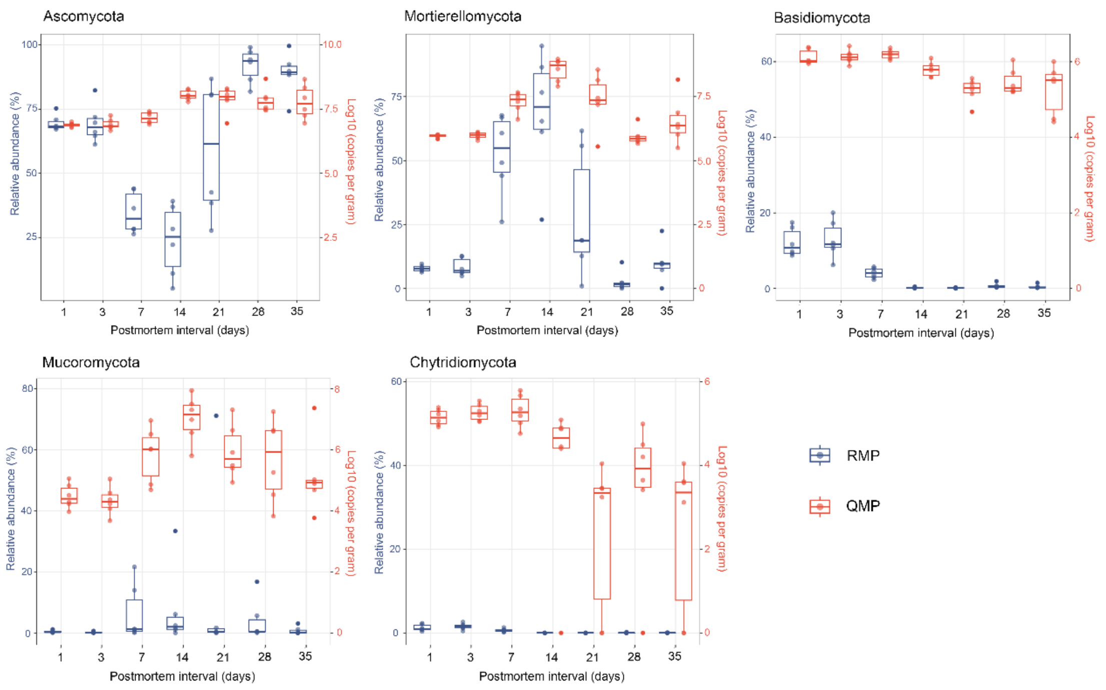

Fig. S4 A boxplot illustrates the comparisons of absolute and relative abundance trends of dominant fungal phyla in grave soil samples. The red indicates absolute abundance, while the blue denotes relative abundance.

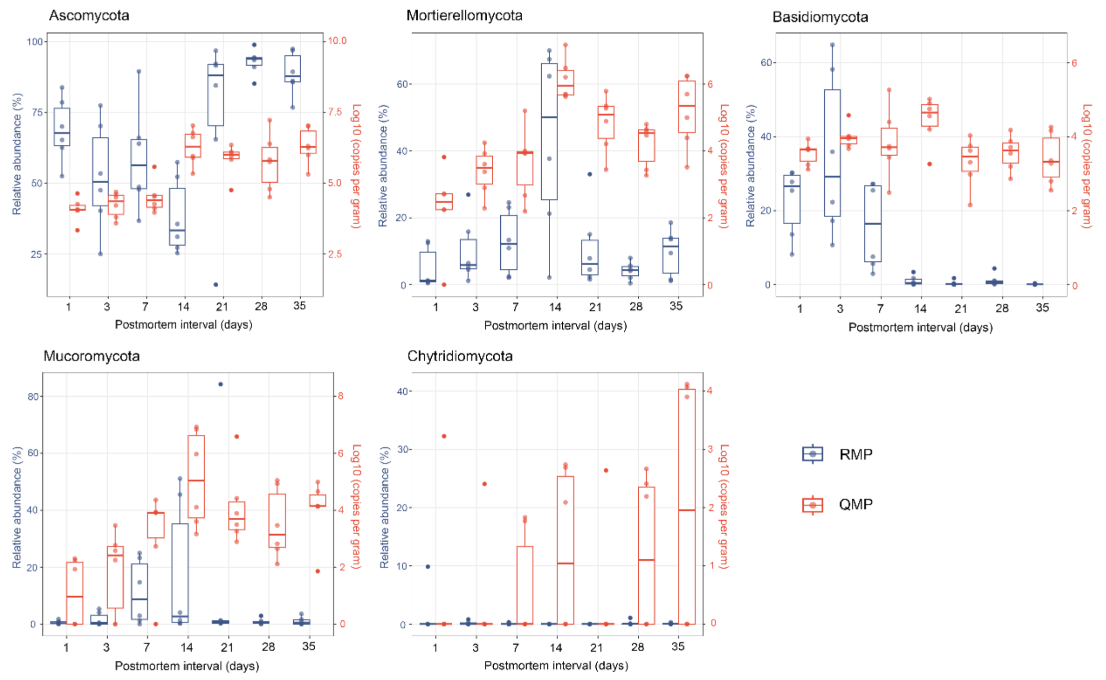

Fig. S5 A boxplot illustrates the comparisons of absolute and relative abundance trends of dominant fungal phyla in tissue samples. The red indicates absolute abundance, while the blue denotes relative abundance.

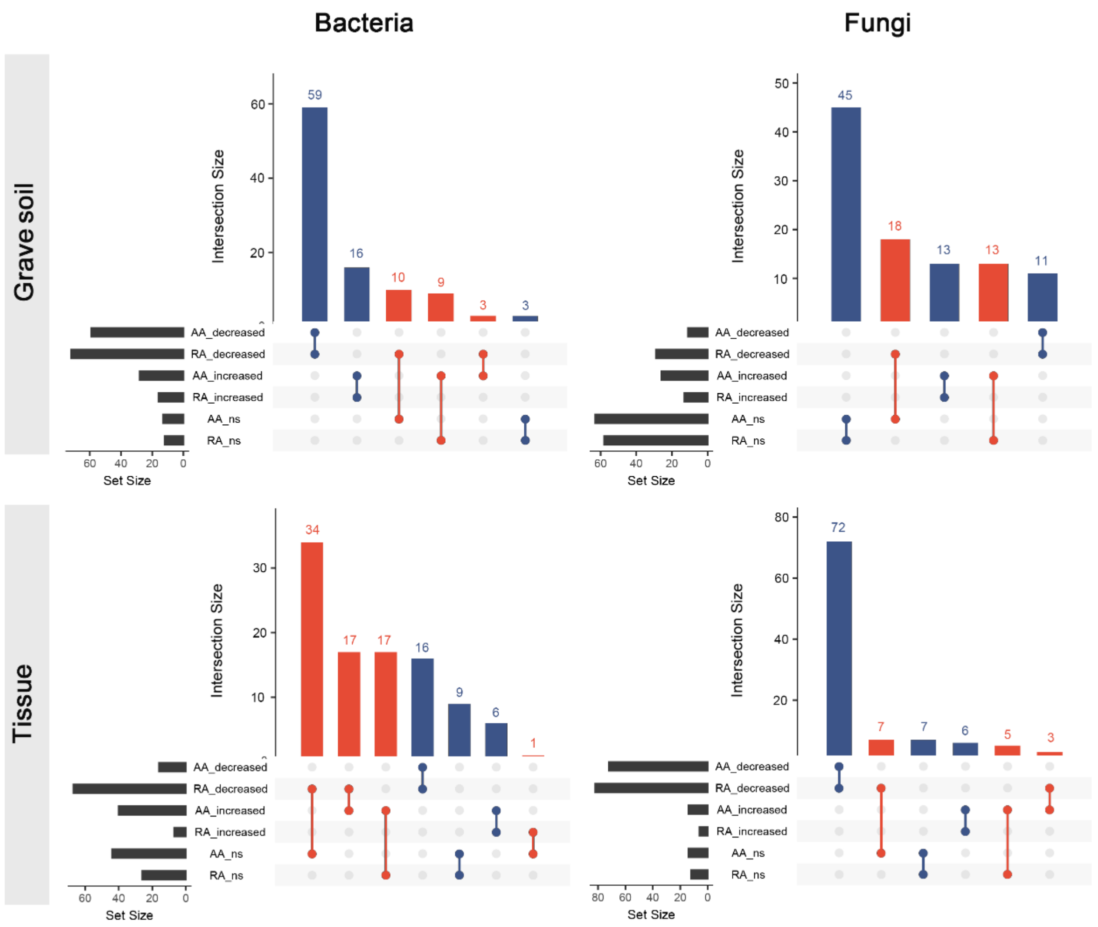

Fig. S6 Upset charts are employed to illustrate the differences in changes of the absolute abundance and relative abundance of the main bacterial and fungal genera in the grave soil and tissue. The differences between samples collected before day 7 and those collected after day 14 were tested using the Wilcoxon rank-sum test with Benjamini-Hochberg correction for the top 100 genera. AA represents absolute abundance and RA represents relative abundance in the figure.

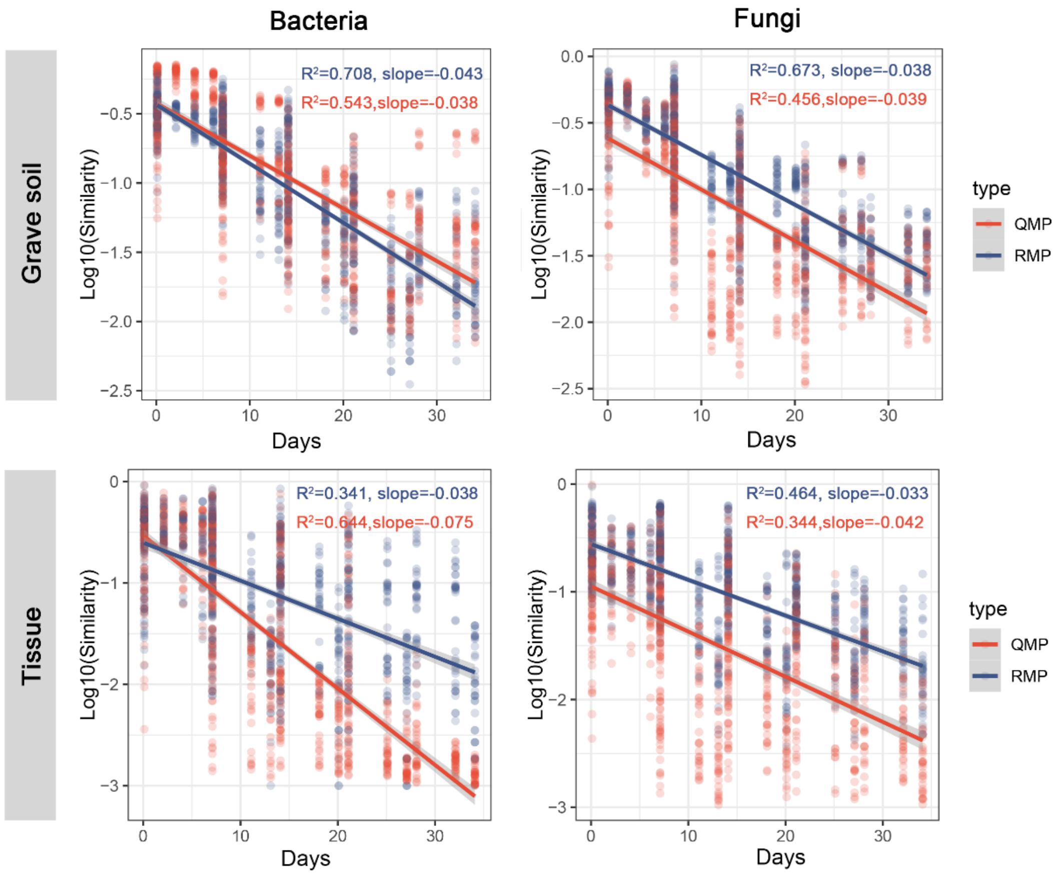

Fig. S7 Comparisons of the succession rates of microbial communities between absolute abundance and relative abundance based on a time-decay relationship between microbial communities and postmortem intervals.

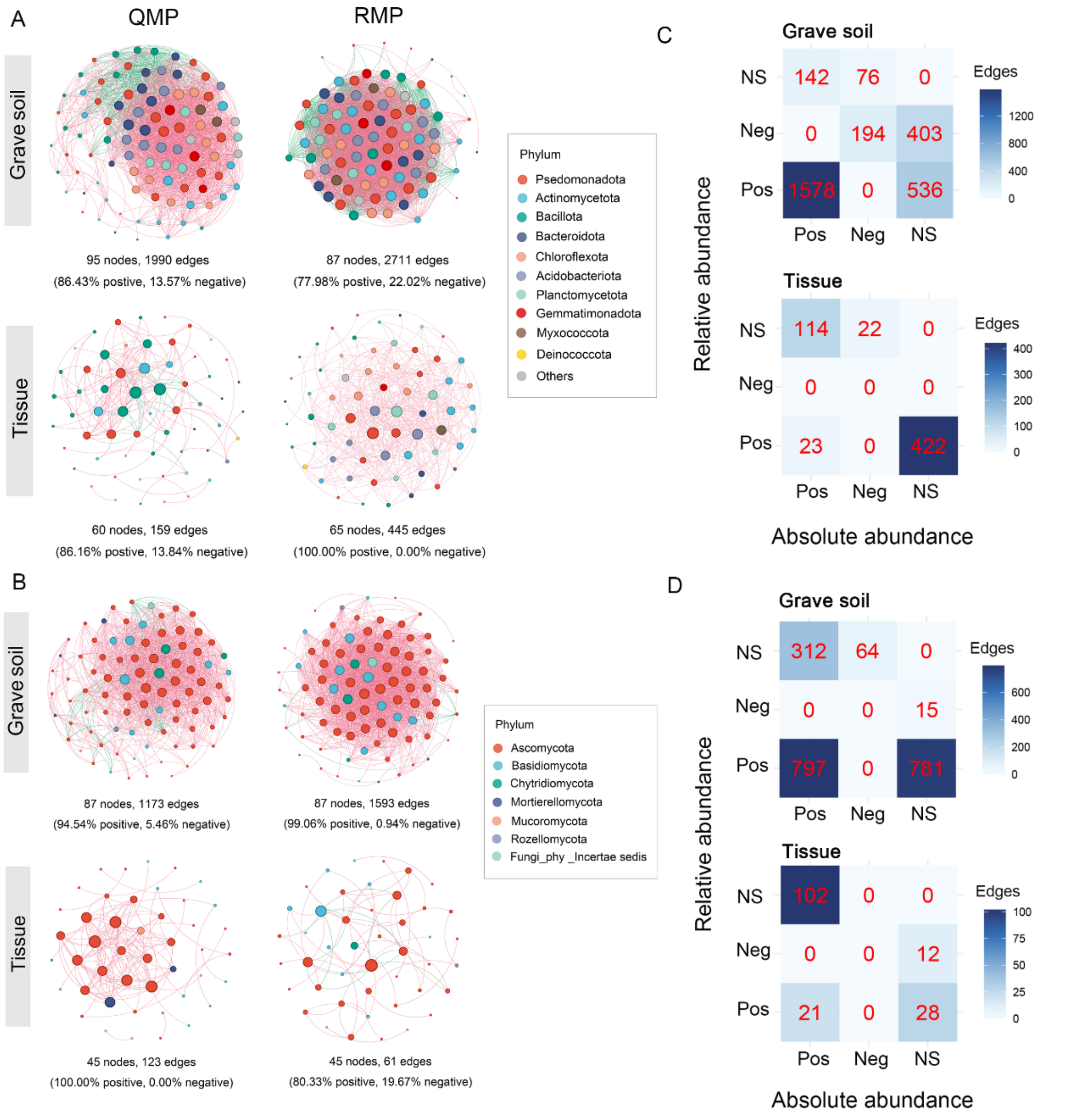

Fig. S8 Comparisons of the co-occurrence relationships between the abundant taxa based on absolute abundance and relative abundance in bacteria (A and C) and fungi (B and D) in grave soil and tissue of carcasses. A and B demonstrate the microbial co-occurrence networks constructed using absolute abundance and relative abundance of abundant taxa. C and D are confusion matrices to compare the consistency the co-occurrence relationships between the absolute abundance and relative abundance.

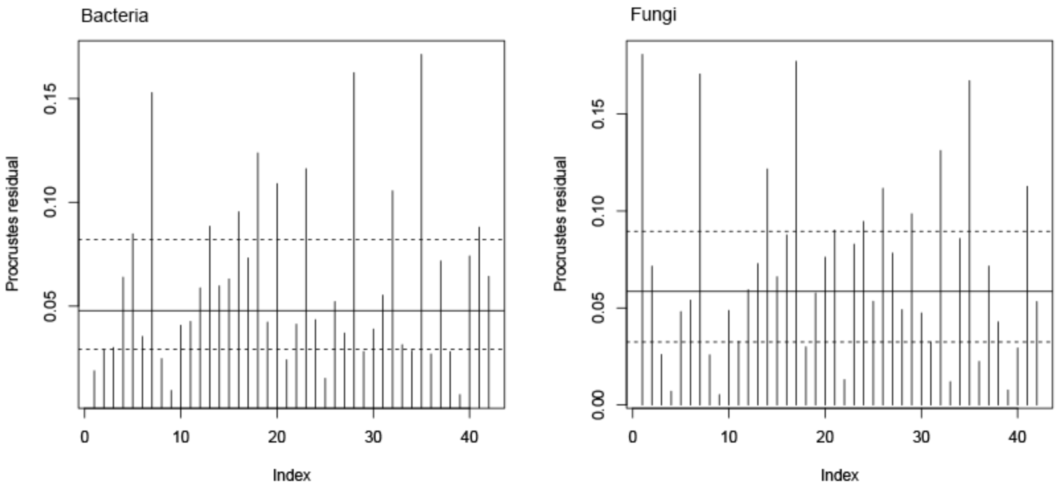

Fig. S9 Bar charts of procrustes residuals.

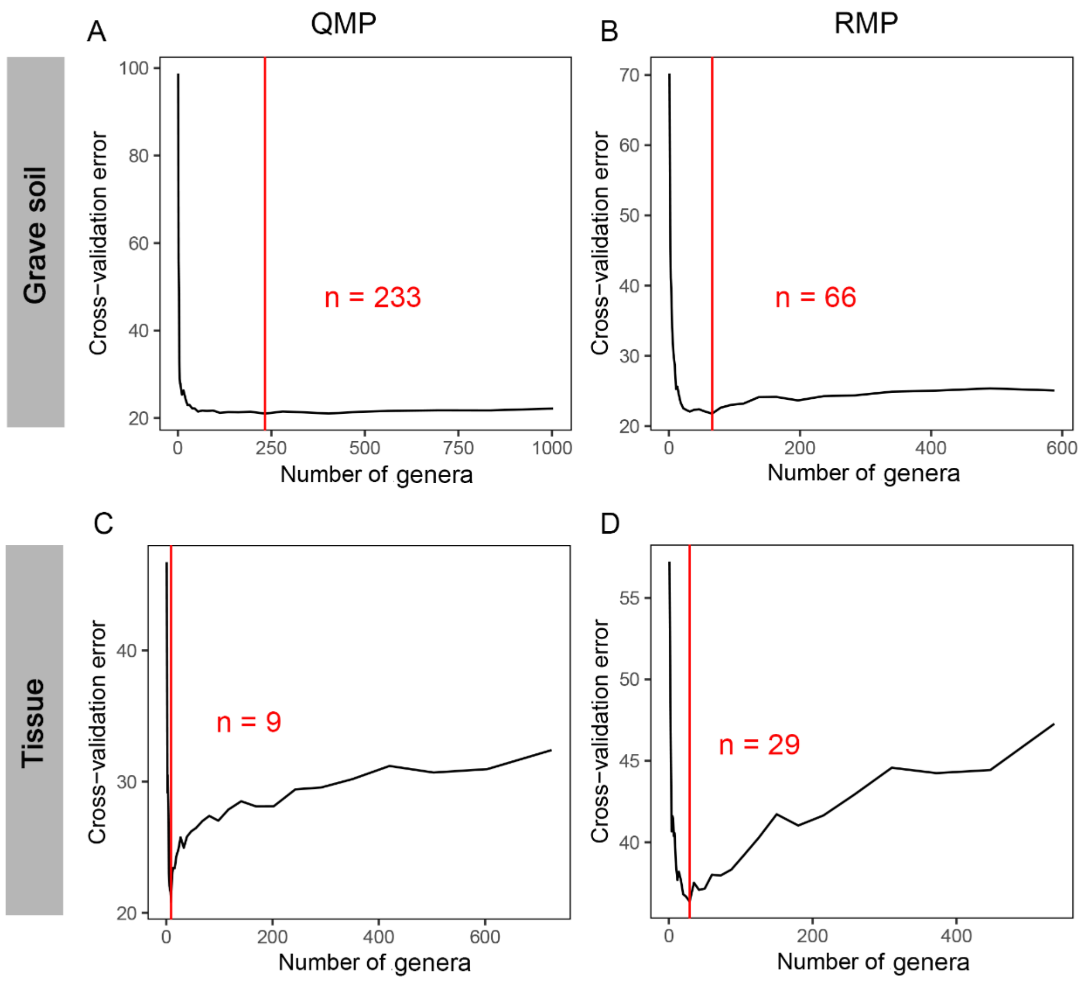

Fig. S10 The optimal sets of bacterial biomarkers in grave soil and tissue for predicting PMI were identified by applying random forest algorithm based on minimum value of 10-fold cross-validation error.

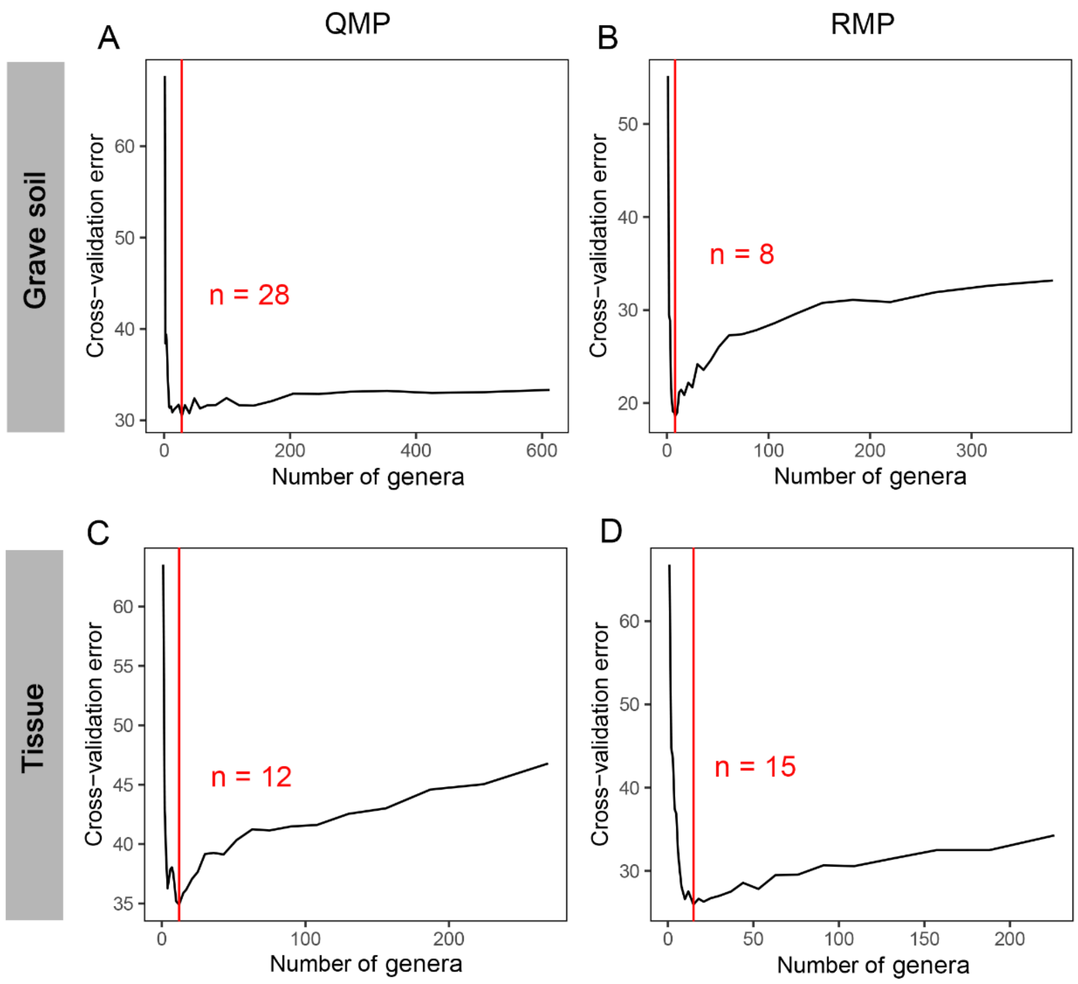

Fig. S11 The optimal sets of fungal biomarkers in grave soil and tissue for predicting PMI were identified by applying random forest algorithm based on minimum value of 10-fold cross-validation error.

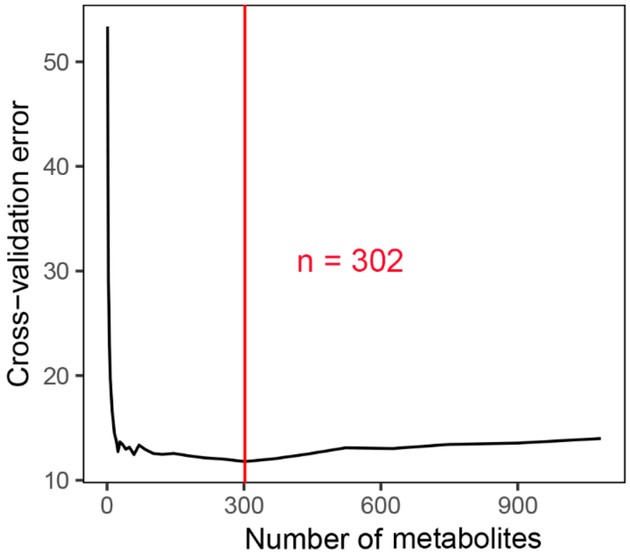

Fig. S12 The optimal sets of metabolic biomarkers in tissue for predicting PMI were identified by applying random forest algorithm based on minimum value of 10-fold cross-validation error.

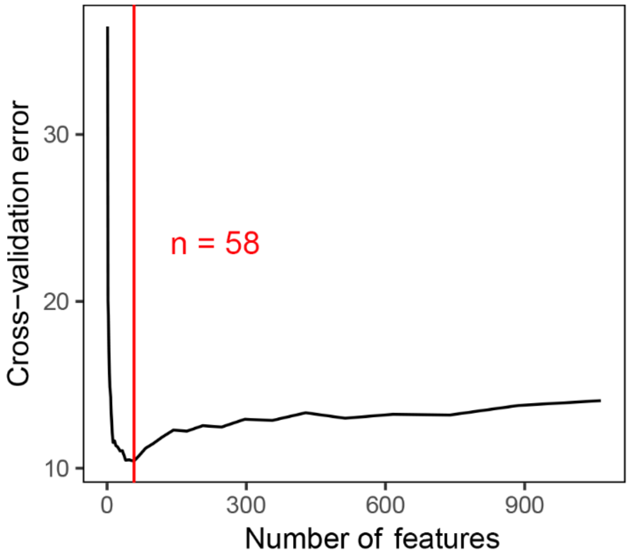

Fig. S13 The optimal sets of multi-omics biomarkers in tissue for predicting PMI were identified by applying random forest algorithm based on minimum value of 10-fold cross-validation error.

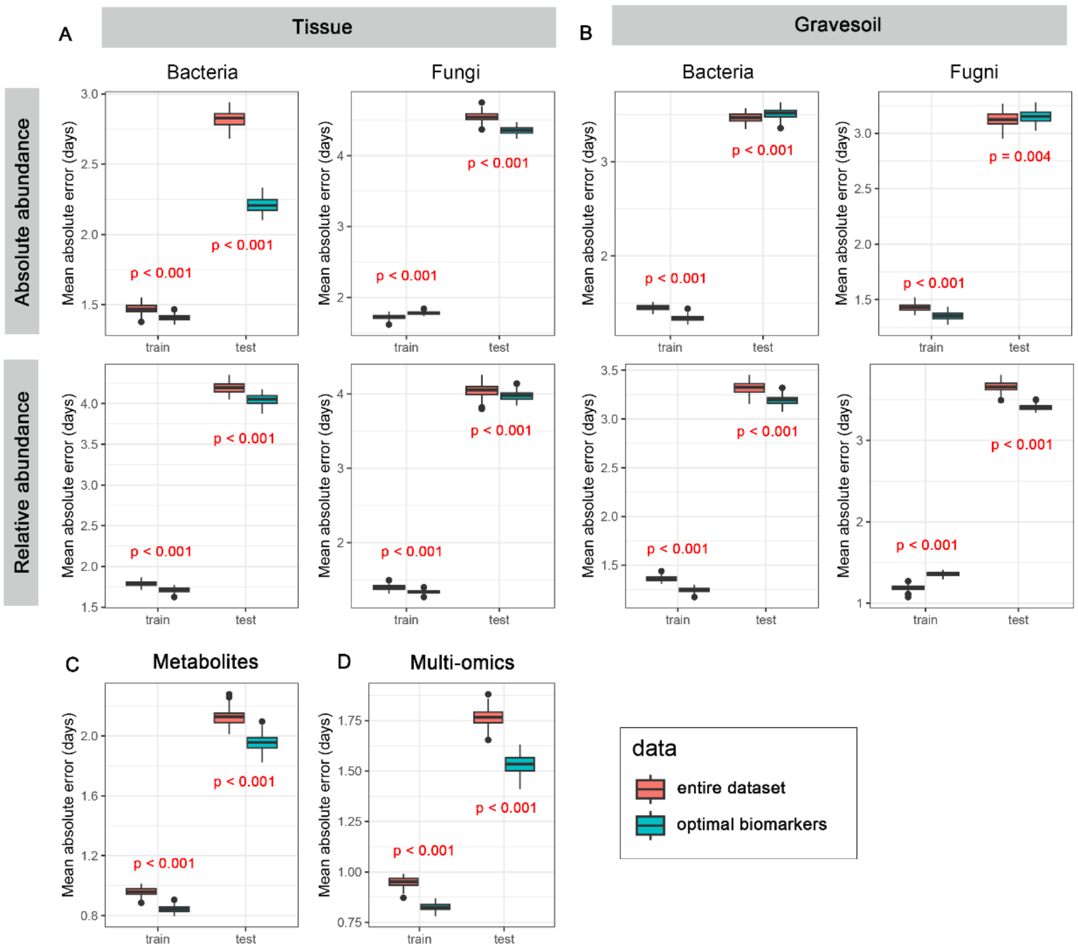

Fig. S14 Comparison of mean absolute errors (MAE) for PMI prediction models constructed using optimal biomarkers versus the entire dataset.

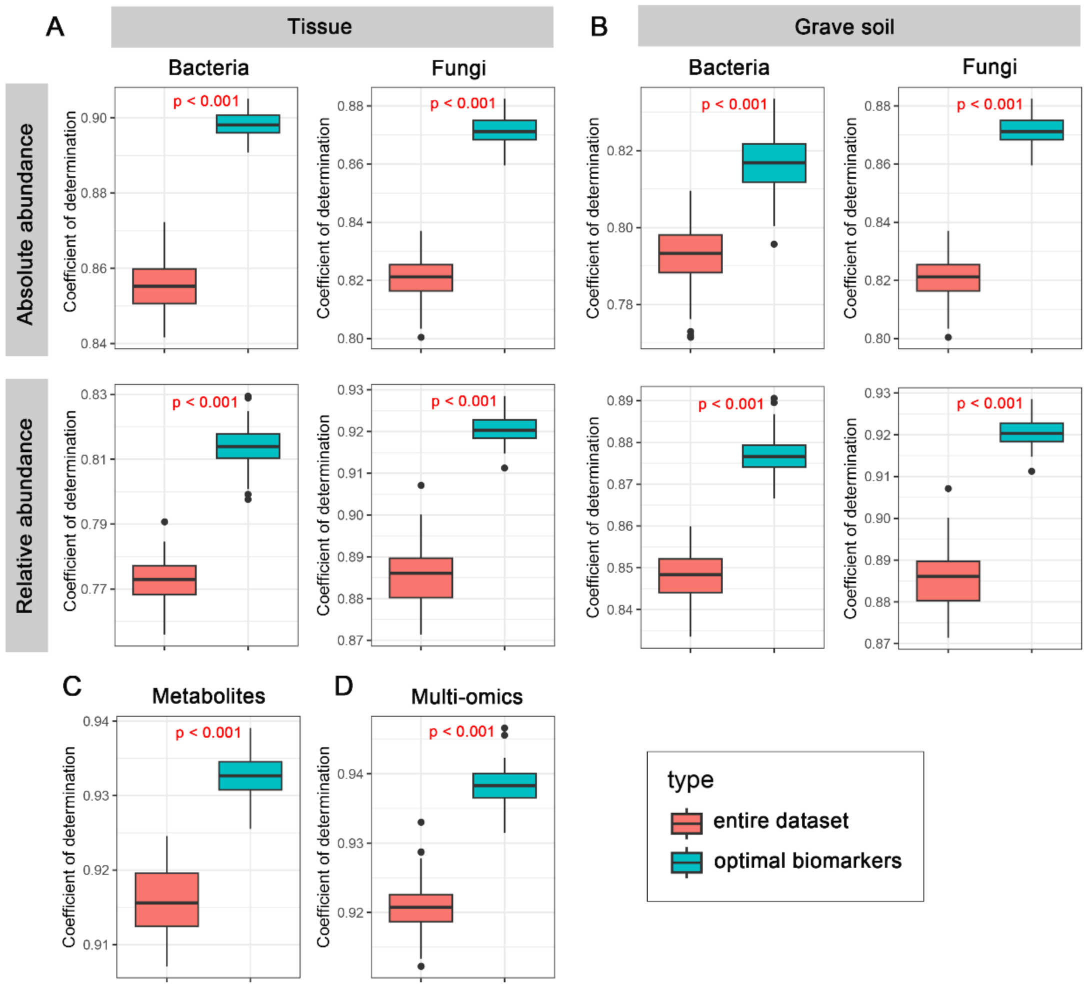

Fig. S15 Comparison of coefficient of determination (R^2) for PMI prediction models constructed using optimal biomarkers versus the entire dataset.

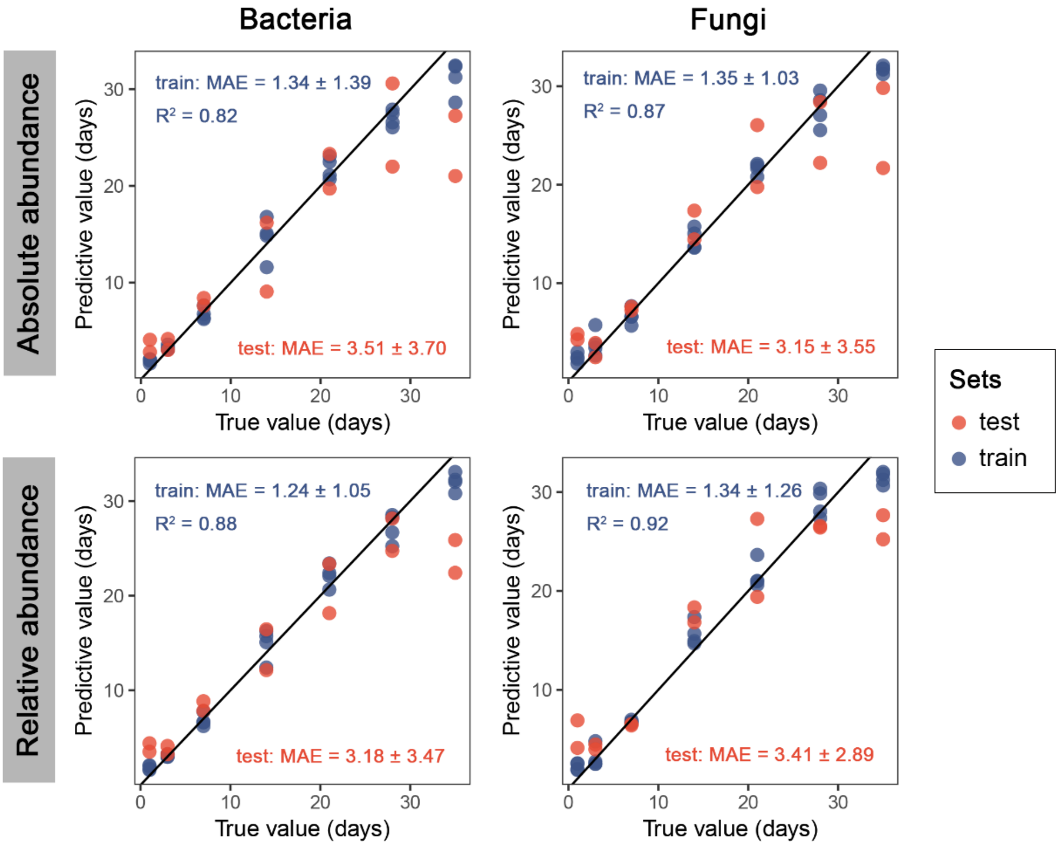

Fig. S16 The predictive accuracies of PMI estimation performed with optimal biomarkers in grave soil.

Table S1 The annotation information of metabolites.

Table S2 Parameters of the microbial co-occurrence networks in tissue of carcasses.

Parameters	Average degree	Average path length	Diameter	Density	Modularity
value	5.90	3.05	8.57	0.03	0.52

Table S3 Biomarker sets were optimized via 10-fold cross-validation with five repetitions and ranked based on the values of the increase in mean decrease accuracy (IncMSE) in the random forest model.

Table S4 Information on sample weight and DNA quantity.

Table S5 The standard curve for each sample, y represents log10 transformed microbial gene copies, and x represents log10 transformed microbial read counts, respectively.
